# An experimental medicine protocol for exploring the haemodynamic effects of dual agonism at the glucagon‐like peptide‐1 and glucagon receptor in healthy subjects

**DOI:** 10.1002/bcp.70282

**Published:** 2025-09-30

**Authors:** James Goodman, Victoria E. Parker, Carmel M. McEniery, Giovanni Di Stefano, Annette Hubsch, Evangelia Vamvaka, Jo Helmy, Fotini Kaloyirou, Navazh Jalaludeen, Peter Barker, Lutz Jermutus, Joseph Cheriyan, Philip Ambery, Ian B. Wilkinson

**Affiliations:** ^1^ Division of Experimental Medicine and Immunotherapeutics, Department of Medicine University of Cambridge UK; ^2^ Clinical Pharmacology Department Cambridge University Hospitals NHS Foundation Trust UK; ^3^ Research and Early Development, Cardiovascular, Renal and Metabolism, BioPharmaceuticals R&D, AstraZeneca Cambridge UK; ^4^ Clinical Project Management, Vendor Partnerships, IQVIA Reading UK; ^5^ NHS Blood and Transplant Clinical Trials Unit Cambridge UK; ^6^ NIHR Cambridge BRC Core Biochemical Assay Laboratory, Cambridge University Hospitals NHS Foundation Trust Cambridge UK; ^7^ Cardiovascular Trials Office, Cambridge Clinical Trials Unit Cambridge University Hospitals NHS Foundation Trust Cambridge UK; ^8^ Late‐stage Development, Cardiovascular, Renal and Metabolism, BioPharmaceuticals R&D, AstraZeneca Gothenburg Sweden

**Keywords:** cardiovascular, dual agonism, exenatide, GLP‐1, GLP‐1:glucagon, glucagon, haemodynamics

## Abstract

**Aims:**

Glucagon‐like peptide‐1 (GLP‐1) and glucagon dual receptor agonists are in clinical development for a range of metabolic conditions, including type 2 diabetes and obesity. The cardiovascular actions at these receptors are well studied, but less is known about their combination. The aim was to explore the acute haemodynamic effects of dual agonism at the GLP‐1 and glucagon receptor.

**Methods:**

Healthy male participants attended randomized, saline‐controlled intravenous infusion studies using glucagon (low, 25 ng/kg/min), glucagon (high, 50 ng/kg/min), exenatide (loading dose 50 ng/min for 30 min then 25 ng/min) and exenatide:glucagon co‐infusion for 120 min in Part A (glucagon dose‐comparison study) and 60 min in Part B (dual‐agonism study).

**Results:**

In Part A (n = 7, median age 21 years, interquartile range 21‐32 years), glucagon (high) increased heart rate by 11 beats per minute (bpm) (95% confidence interval [CI] 4‐17 bpm, *P* < .01). In Part B (n = 12, median age 24 years, interquartile range 22‐26 years), exenatide increased heart rate by 4 bpm (95% CI 2‐6 bpm, *P* < .001). Glucagon (low) increased heart rate by 4 bpm (95% CI 1‐7 bpm, *P* < .001). Co‐infusion of glucagon (low) and exenatide increased heart rate by 7 bpm (95% CI 4‐9 bpm, *P* < .001) and the rate pressure product by 793 mmHg*bpm (95% CI 460‐1127 mmHg*bpm, *P* < .001). There were no differences in cardiac output, blood pressure or heart rate variability.

**Conclusions:**

In healthy males, exenatide and glucagon co‐infusion acutely increases the rate pressure product, an indirect measure of cardiac work. This increase is driven by an increase in heart rate, rather than any change in systolic blood pressure.

**What is already known about this subject**

- Glucagon‐like peptide‐1 (GLP‐1) receptor agonists reduce cardiovascular events in adults with type 2 diabetes, obesity and chronic kidney disease.
- GLP‐1 and glucagon dual receptor agonists are in clinical development for chronic metabolic conditions including type 2 diabetes, obesity and metabolic dysfunction associated steatohepatitis.

**What this study adds**

- Co‐infusion of exenatide (a licensed GLP‐1 receptor agonist) and glucagon acutely increases the rate pressure product (heart rate multiplied by systolic blood pressure).
- The increase in the rate pressure product is driven by an acute increase in heart rate, rather than any change in systolic blood pressure.
- The short‐term increase in the rate pressure product seen with co‐infusion may be consistent with long‐acting dual receptor agonists in the initial period after starting therapy.

## 1 INTRODUCTION

Native glucagon‐like peptide‐1 (GLP‐1) (7‐36) amide is secreted by intestinal L cells in response to a meal. In addition to its metabolic action in regulating glucose homeostasis, GLP‐1 has various effects on the cardiovascular system, either via activation of the GLP‐1 receptor or following DPP‐4 mediated cleavage. In contrast to native GLP‐1, licensed GLP‐1 receptor agonists produce their actions directly through the GLP‐1 receptor and are licensed for the treatment of type 2 diabetes and obesity, where they have proven cardiovascular benefits.^1^

Native GLP‐1 infusion increases heart rate when infused into healthy volunteers,^2^, ^3^, ^4^ and patients with type 2 diabetes^5^ and heart failure.^6^ Likewise, long‐acting GLP‐1 receptor agonists induce a small rise in heart rate by ~1‐10 beats per minute (bpm)^7^ via activation of the sino‐atrial node and activation of the sympathetic nervous system.^8^ Long‐term administration of synthetic GLP‐1 receptor agonists is consistently shown to reduce blood pressure (BP). The GLP‐1 receptor is widely expressed in the heart^9^ and vasculature.^10^ Hearts from transplant patients and deceased organ donors express GLP‐1 mRNA in all four chambers.^11^ GLP‐1 mRNA receptor transcripts are also found in the sino‐atrial node,^9^ and atrial and ventricular cardiomyocytes from normal and ischaemic human hearts.^12^

Glucagon is a peptide hormone secreted by pancreatic alpha‐cells in response to hypoglycaemia. Experimental cardiovascular studies first conducted by Parmley et al in 1968 show that intravenous (IV) glucagon increases cardiac index, mean arterial pressure (MAP), heart rate and maximum left ventricular (LV) pressure in humans during cardiac catheterisation.^13^ Data from the 1970s and 1980s further demonstrate that IV glucagon increases heart rate, BP and cardiac contractility.^14^ Infusion studies have since reproduced these findings on cardiac chronotropy, but the effects on other haemodynamic variables are less clearly defined.^14^

Dual and multi‐agonist synthetic peptides at the GLP‐1 and glucagon receptors are in clinical development as potential new treatments for type 2 diabetes, obesity, chronic kidney disease and metabolic dysfunction‐associated steatohepatitis. Simultaneous targeting of the GLP‐1 and glucagon receptors aims to leverage the beneficial metabolic effects of these receptors to suppress appetite and reduce body weight, whilst simultaneously balancing opposing effects on glucose metabolism. The cardiovascular actions of GLP‐1 and glucagon receptor agonism are well studied but limited data exist on the acute haemodynamic effects of dual receptor agonism. Considering GLP‐1 receptor agonism and glucagon receptor agonism both increase heart rate, one question that was highlighted at the onset of clinical development of dual agonist therapy was whether there may be an acute and significant increase in cardiac work in the early stages of treatment. Drugs which increase cardiac work or cardiac output are generally associated with a worse long‐term prognosis in patients with heart failure and cardiovascular disease.^15^ A study examining the acute haemodynamic effects of GLP‐1:glucagon dual receptor agonism was designed to explore this.

## 2 METHODS

### 2.1 Study design

This was a single‐centre, randomized, placebo‐controlled, exploratory, single‐blinded, physiological, mechanistic study. The protocol received approval from the East Midlands Nottingham 1 Research Ethics Committee (18/EM/0417). The study was registered on **Clinicaltrials.gov** (NCT03835013) and performed according to the principles of the Declaration of Helsinki. Recruitment commenced in February 2019 and completed in September 2021. Recruitment was interrupted in 2020 due to the impact of the COVID‐19 pandemic. Part A was a single‐blinded, dose‐comparison study to assess the tolerability of a 2‐h glucagon infusion in an escalating dose. Part B was a single‐blinded, experimental study to assess the safety, tolerability and non‐invasive haemodynamic effects of dual agonism at the GLP‐1 and glucagon receptor. To simulate the acute haemodynamic effects of GLP‐1 and glucagon receptor dual agonism, exenatide (a licensed GLP‐1 receptor agonist which shares 53% amino acid homology with native GLP‐1^16^) and glucagon were intravenously infused. At the time of protocol development, dual GLP‐1:glucagon receptor agonists were in early‐stage clinical development and therefore the decision was made to use IV agents. Importantly, this has the advantage of being able to study drug effects with 100% bioavailability.

**FIGURE 1:**
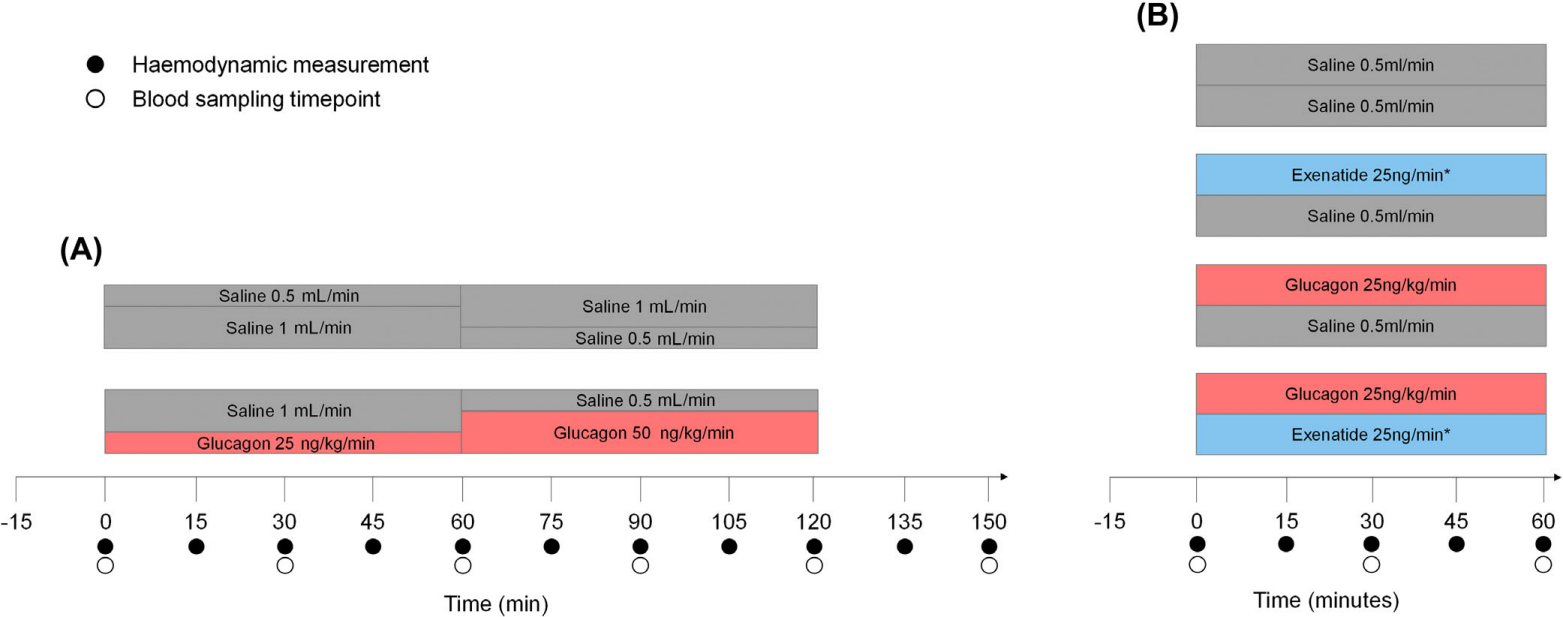
Schematic diagram of (A) Part A and (B) Part B. *50 ng/min for 30 min followed by 25 ng/min.

Using a Latin square block design, participants acted as their own control and received a different infusion at each study visit. Two IV cannulas were sited in the upper limbs on the opposite sides, one for the infusion and the other reserved for blood sampling. Two pairs of infusions consisting of combinations of 0.9% normal saline (Macopharma), glucagon (Novo Nordisk) at concentrations of 25 ng/kg/min (low dose) or 50 ng/kg/min (high dose) and exenatide (50 ng/min loading dose for 30 min then 25 ng/min) were simultaneously infused through a single cannula, via a double lumen extension set, for 120 min (Part A) or 60 min (Part B) at either 0.5 or 1 mL/min (Alaris GH syringe pump, Becton Dickinson) as per the experimental protocol in Figure 1. A number of adverse events, specifically nausea and vomiting, were reported in Part A and accordingly the decision was taken to only infuse the 25 ng/kg/min glucagon dose in Part B to reduce the risk of adverse events and better simulate dual agonist compounds. A low‐sorb infusion line (Alaris, P7000 extension set, Becton Dickinson) was used for all IV infusions. Dose selection was based on previous similar studies.^17^, ^18^, ^19^, ^20^

### 2.2 Participants

The study enrolled non‐smoking, adult male participants between the ages of 18 and 40 years with a body mass index (BMI) of 18‐30 kg/m^2^. Participants attended an initial screening visit where written informed consent was obtained. Participants were determined to be healthy by means of a medical history, physical examination including anthropometric measurements, vital signs, 12‐lead electrocardiogram (ECG) and transthoracic echocardiogram. Those with hypertension, clinically significant heart disease, renal or liver impairment, or diabetes were excluded.

### 2.3 Measurements

Participants were studied in the resting supine position following an 8‐h overnight fast. They were asked to refrain from alcohol and caffeine consumption for 12 h and to avoid non‐steroidal anti‐inflammatory drugs for 24 h before study visits. Strenuous exercise was avoided for 12 h prior to infusion visits. Visits were carried out in a quiet, temperature‐controlled room. Haemodynamic measurements were taken every 15 min and blood samples every 30 min (Accu‐Check glucose monitor, Roche) (Figure 1).

Cardiac output, stroke volume and heart rate were measured in duplicate over an average of 60‐s time intervals using the Cheetah Starling Stroke Volume Haemodynamic Monitoring System (Cheetah Medical, a validated, non‐invasive monitoring technique based on thoracic bioreactance.^21^ Peripheral vascular resistance was calculated as MAP divided by cardiac output, and then multiplied by 80 to convert arbitrary units to dynes/s/cm^5^ as per previously published research.^22^ Brachial systolic BP, brachial diastolic BP, central systolic BP, central diastolic BP, MAP, heart rate and augmentation index were measured using SphygmoCor XCEL (AtCor Medical). The rate pressure product, an indirect measure of cardiac work, was calculated by multiplying systolic BP by heart rate. Heart rate variability was measured using the SphygmoCor Device (AtCor Medical). Using a three‐lead ECG, a continuous, short‐term recording of heart rate was made over 5 min to provide an indication of autonomic nervous system activity. Fast Fourier transform of the ECG signal was used to derive the low frequency to high frequency power ratio (normalized for total power).^23^

### 2.4 Assays

Blood samples in EDTA tubes were collected for analysis of total GLP‐1, total active GLP‐1, glucagon, glucose‐dependent insulinotropic polypeptide (GIP) and peptide YY. Heparin lithium tubes analysed insulin, C‐peptide, free fatty acids and triglycerides. All venous blood was collected in chilled 5‐mL syringes containing 20 μL of dipeptidyl peptidase‐4 inhibitor (DPP4i) (Merck). Blood was then transferred into a chilled 2.6‐mL EDTA tube containing 100 μL of aprotinin 10 000 Kallikrein Inactivator Units/mL (Trasylol, Nordic Pharma). All syringes and blood tubes were stored in wet ice prior to sampling. Aprotinin is an enzyme inhibitor that prevents glucagon degradation and DPP4i inhibits degradation of GLP‐1. Samples were collected in chilled lithium heparin tubes. Blood tubes were placed immediately on wet ice, centrifuged at 4 °C, 3005 xg for 10 min, snap frozen on dry ice and stored at less than −40 °C until analysis.

### 2.5 Statistical analysis

Data were analysed using SPSS (version 28, IBM). No formal power calculations were used as this was an exploratory study with a sample size based on similar experimental pilot studies.^18^, ^19^, ^20^ Cardiovascular data were analysed using a two‐way repeated measures analysis of variance (ANOVA) with post hoc testing (paired *t*‐test). Metabolic data were analysed using a two‐way repeated measures ANOVA with post hoc testing (paired *t*‐test with Bonferroni correction). Area under the curve (AUC) data were analysed using a one‐way ANOVA and post hoc testing (paired *t*‐test). Heart rate variability data (non‐parametric) were plotted using a forest plot and analysed using the Wilcoxon signed rank test. Data are expressed as mean ± standard error of the mean (SEM), or percentages. Line and bar graphs represent the mean and error bars represent SEMs. A *P* value <.05 was deemed significant for all statistical analyses.

## 3 RESULTS

**FIGURE 2:**
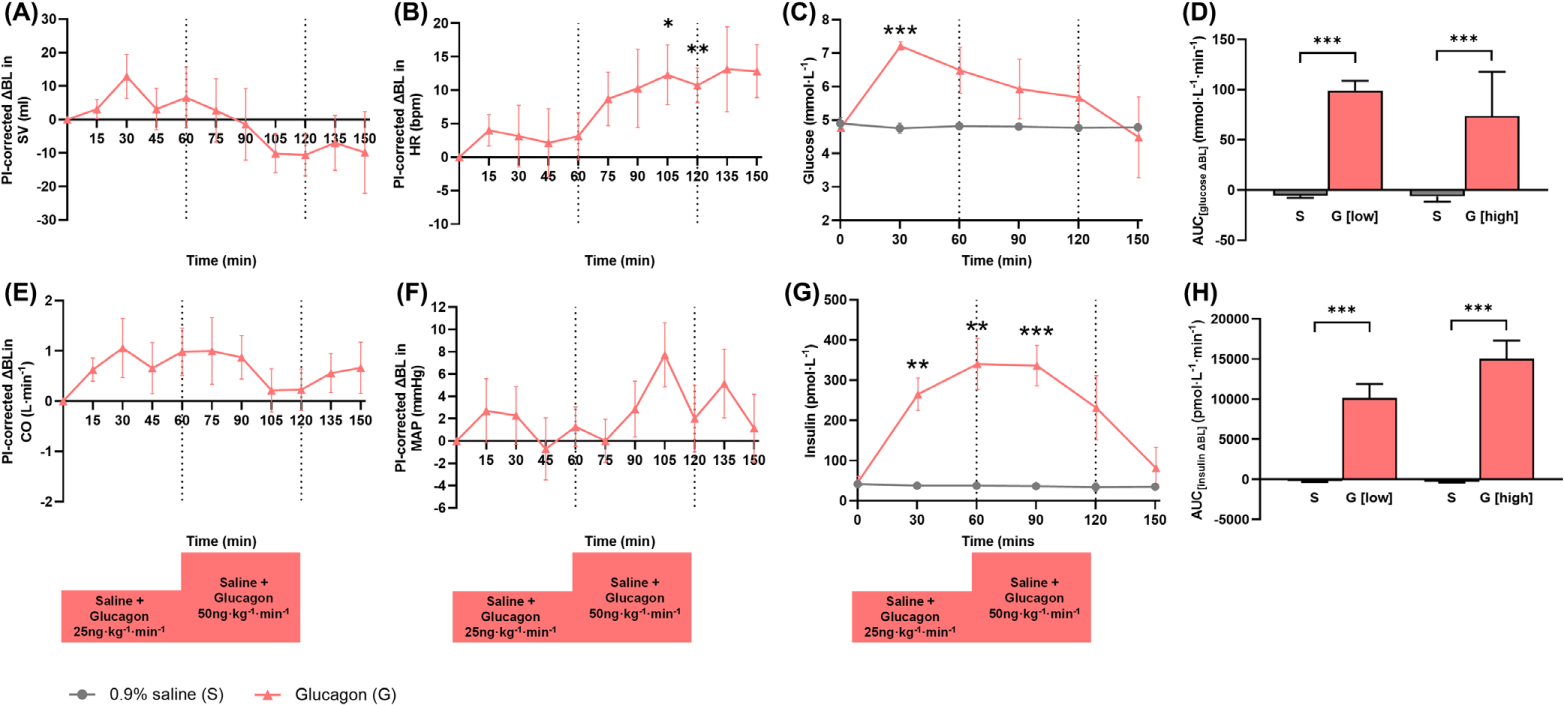
The haemodynamic and metabolic effects of an intravenous saline and glucagon infusion over 120 min (Part A): (A) stroke volume, (B) heart rate, (C) plasma glucose, (D) plasma glucose, change from baseline presented as the area under the curve (0‐120 min), (E) cardiac output, (F) mean arterial pressure,(G) insulin and (H) insulin, change from baseline, presented as the area under the curve (0‐120 min). Data are unadjusted means ± standard error of the mean. BL, baseline; AUC, area under the curve; CO, cardiac output; G, glucagon; HR, heart rate; pl‐corrected, placebo corrected; MAP, mean arterial pressure; S, saline; SV, stroke volume. *P < .05, **P < .01, ***P < .001.

Seven healthy males with a median age of 21 years (interquartile range 21‐32 years) and BMI 22 kg/m^2^ completed Part A (Table 1). Data are presented in Figure 2 and Supporting Information Figures S1‐S6. Unless differently stated, all statistical comparisons are against saline as control. Glucagon (high dose, 50 ng/kg/min) significantly increased heart rate at 120 min by 11 bpm (95% confidence interval [CI] 4‐17 bpm, *P* < .01) (Figure 2B). No differences in cardiac output, brachial BP, central BP, MAP, peripheral vascular resistance, augmentation index or heart rate variability were observed between saline and glucagon. The metabolic results show glucagon (low and high) transiently increased blood glucose and plasma insulin (AUC, *P* < .001 for both) (Figure 2C,D,G,H).

**TABLE 1:** Baseline characteristics for Parts A and B.

	Part A	Part B
Number	7 males	12 males
Age (years)	21 (21‐32)	24 (22‐26)
Age range (years)	19‐37	20‐33
Height (cm)	183 (166‐186)	182 (173‐186)
Weight (kg)	72 (51‐89)	77 (59‐88)
BMI (kg/m^2^)	22 (19‐26)	22 (20‐27)
Body fat (%)	15 (10‐24)	11 (9‐15)
SBP (mmHg)	120 (118‐120)	118 (113‐125)
DBP (mmHg)	71 (64‐76)	66 (63‐68)
HR (bpm)	76 (65‐90)	61 (55‐75)
HbA1C (mmol/mol)	35 (34‐38)	33 (31‐34)

*Note*: Data are median (interquartile range). Abbreviations: BMI, body mass index; DBP, diastolic blood pressure; HR, heart rate; MAP, mean arterial pressure; SBP, systolic blood pressure.

**FIGURE 3:**
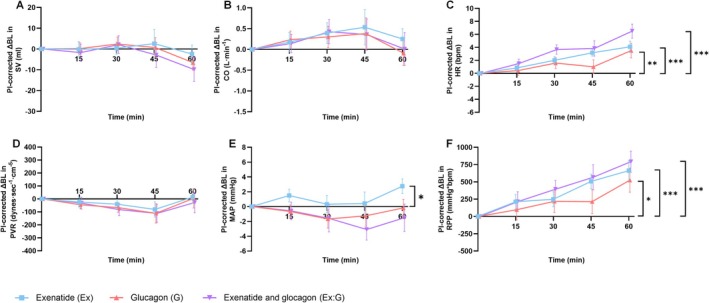
The haemodynamic effects of an intravenous infusion of saline, glucagon, exenatide and combination exenatide:glucagon over 60 min (Part B): (A) stroke volume, (B) cardiac output, (C) heart rate, (D) peripheral vascular resistance, (E) mean arterial pressure and (F) rate pressure product. Data are unadjusted means ± standard error of the mean. BL, baseline; AI, augmentation index; AUC, area under the curve; CO, cardiac output; HR, heart rate; pl‐corrected, placebo corrected; MAP, mean arterial pressure; PVR, peripheral vascular resistance; RPP, rate pressure product; SV, stroke volume. *P < .05, **P < .01, ***P < .001.

**FIGURE 4:**
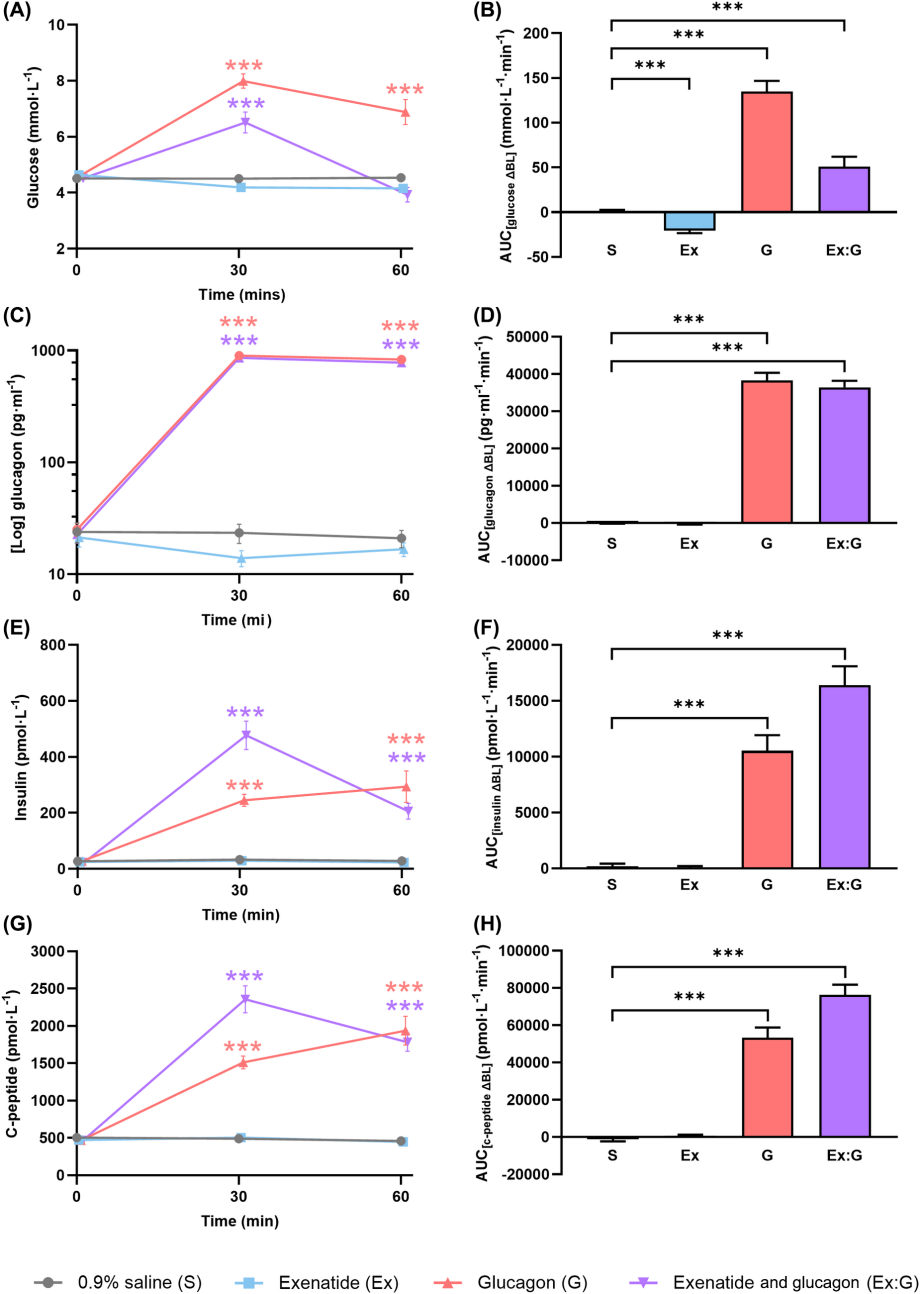
The metabolic effects of an intravenous infusion of saline, glucagon, exenatide and combination exenatide:glucagon over 60 min (Part B): (A) plasma glucose, (B) plasma glucose, change from baseline presented as the area under the curve (0‐60 min), (C) glucagon (total of infused, synthetic, glucagon plus native glucagon), (D) glucagon, change from baseline presented as the area under the concentration‐time curve (0‐60 min), glucagon equals the total of infused (synthetic) glucagon plus native glucagon, (E) insulin, (F) insulin, change from baseline presented as the area under the curve (0‐60 min), (G) C‐peptide and (H) C‐peptide, change in baseline presented as the area under the curve (0‐60 min). Data are unadjusted means ± standard error of the mean. BL, baseline; AUC, area under the curve; ex, exenatide; G, glucagon; S, saline. *P < .05, **P < .01, ***P < .001.

Twelve healthy males with a median age of 24 years (interquartile range 22‐26 years) and BMI 22 kg/m^2^ completed Part B (Table 1). Data are presented in Figures 3 and 4 and Supporting Information Figures S7‐S15. Glucagon low dose (25 ng/kg/min) was infused in Part B. When infused in isolation, both glucagon and exenatide caused a small but significant increase in heart rate over time (two‐way ANOVA time × drug, *P* < .001) (Figure 3C). At 60 min, mean heart rate was +4 bpm (95% CI 2‐6 bpm, *P* < .001) with exenatide and +4 bpm (95% CI 1‐7 bpm, *P* < .001) with glucagon, compared with saline. Exenatide:glucagon co‐infusion caused a greater increase in heart rate, such that the mean heart rate was +7 bpm (95% CI 4‐9 bpm, *P* < .001) in the combination infusion at 60 min. Exenatide increased MAP by +3 mmHg (95% CI 1‐5 mmHg, *P* < .05) at 60 min (Figure 3E). At 60 min exenatide:glucagon increased the rate pressure product by 793 mmHg*bpm (95% CI 460‐1127 mmHg*bpm, *P* < .001) (Figure 3F). In isolation, glucagon and exenatide increased the rate pressure product by 526 mmHg*bpm (95% CI 142‐909 mmHg*bpm, *P* < .05) and 663 mmHg*bpm (95% CI 346‐980 mmHg*bpm, *P* < .001), respectively. There were no significant overall differences in stroke volume, cardiac output, brachial BP, central BP, peripheral vascular resistance and heart rate variability between saline and any of the infusion arms. Glucagon and exenatide:glucagon co‐infusion caused an increase in plasma glucose (AUC, *P* < .001) (Figure 4A,B). In isolation, exenatide induced a mild, but significant, decrease in plasma glucose (AUC, *P* < .001). Both the glucagon and combination infusions increased plasma insulin and C‐peptide (AUC, *P* < .001 for both) (Figure 4E‐H). The glucagon and combination infusions significantly reduced free fatty acids (AUC, *P* < .001) (Supporting Information Figures S13A and S13B). There were no differences between total GLP‐1, total active GLP‐1, GIP, peptide YY or triglycerides between any of the infusions.

### 3.1 Adverse events

Ten adverse events were recorded in Part A and 10 in Part B. In Part A, hypoglycaemia (defined as a blood glucose <4.0 mmol/L) occurred six times, all in the glucagon infusion arm. Four of these hypoglycaemic episodes were symptomatic (three with nausea and one with vomiting). Asymptomatic hypoglycaemia occurred 10 times in Part B (twice in the glucagon infusion arm and eight times in the exenatide:glucagon co‐infusion arm). Hypoglycaemia was treated with IV dextrose until blood glucose was >4.0 mmol/L. There were no serious adverse events.

## 4 DISCUSSION

The main findings of this study were that in healthy male participants glucagon and exenatide infusions, in isolation, increased heart rate by ~4 bpm at 60 min (*P* < .001). Co‐infusion led to a greater increase in heart rate of ~7 bpm (*P* < .001). Exenatide infusion increased MAP by ~3 mmHg (*P* < .05). Glucagon, exenatide and exenatide:glucagon co‐infusion significantly increased the rate pressure product by 526 mmHg*bpm (*P* < .05), 663 mmHg*bpm (*P* < .001) and 793 mmHg*bpm (*P* < .001), respectively. Co‐infusion did not markedly impact any other haemodynamic variable. Glucagon and exenatide:glucagon co‐infusion increased plasma glucose, glucagon, insulin and C‐peptide (*P* < .001). Exenatide decreased plasma glucose (*P* < .001).

### 4.1 GLP‐1

Our data align with previous studies showing that IV exenatide increases heart rate through a number of established mechanisms, including direct activation of pacemaker cells of the sino‐atrial node.^24^ Activation of the autonomic system is reported to entail chronotropic effects,^25^ but data from large animal models^24^ and humans^3^ fail to support GLP‐induced activation of the sympathetic nervous system. In agreement, GLP‐1‐related changes in sympathetic activity were not observed in this study based on heart rate variability. The small increase in heart rate (~4 bpm) had no significant effect on cardiac output. It is possible that compensatory physiological changes in stroke volume and peripheral vascular resistance were too small to detect in this pilot study. Similar haemodynamic patterns in heart rate (significant increase), stroke volume (no significant change), cardiac output (no significant change) and peripheral vascular resistance (no significant change) were observed in the other infusion arms. The rate pressure product significantly increased in all three infusion arms and this is expanded on below. Intravenous exenatide did cause a small, but significant, 3 mmHg increase in MAP in our infusion study, which broadly aligns with other studies of IV exenatide (Supporting Information Table S1). By contrast, other GLP‐1 receptor agonists have been reported to reduce systolic BP by ~0.7‐4.6 mmHg.^26^ This may suggest that exenatide has differential effects on the cardiovascular system compared with other GLP‐1 receptor agonists, although we cannot determine if the chronic effects on BP may be different to our findings in this short‐term study. Mechanisms relating to the contrasting effects of acute infusions of GLP‐1 receptor agonists (either native GLP‐1 or exenatide) versus the long‐term effects of licensed GLP‐1 receptor agonists on BP are not fully understood. Blood pressure reductions occur early (after 2 weeks) with prolonged use of GLP‐1 receptor agonists, suggesting that weight loss alone is not the only driver for BP reductions. Additional mechanisms which decrease BP include arterial and smooth muscle activation of GLP‐1 receptors, improvements in vascular endothelial function, nitric oxide induced vasodilation and inhibition of the renin‐angiotensin‐aldosterone system (natriuresis).^27^ The timescale and the relative contribution of these mechanisms to overall BP reduction is somewhat unclear, although likely accounts for at least some of the observed differences in BP between acute infusions and chronic treatment with GLP‐1 receptor agonists.

### 4.2 Glucagon

Glucagon demonstrated an increase in heart rate that is consistent with previous data.^17^, ^18^, ^28^ No change in stroke volume, cardiac output or MAP was observed and this aligns with other low‐dose infusion studies.^29^ Mechanistically, it is unclear if these chronotropic effects result from any direct effects on the myocardium given that the glucagon receptor (and mRNA transcripts) is absent from the sino‐atrial node, atria and ventricles of multiorgan donors and explanted hearts.^30^ One mechanism for an increase in heart rate is via glucagon‐induced activation of myocardial GLP‐1 receptors.^28^ Glucagon and native GLP‐1 share 47% amino acid homology plus overlapping binding sites, enabling glucagon to bind to the GLP‐1 receptor with low affinity.^31^, ^32^ Glucagon‐induced activation of the sympathetic nervous system has been shown to potentiate the chronotropic effects by stimulating the hypophysis‐hypothalamus‐adrenal axis^33^ and through increased catecholamine release.^34^ However, based on heart rate variability, we found no evidence of increased sympathetic activity in this study and it is possible these effects are only seen with higher doses of glucagon.

### 4.3 Dual agonism

Exenatide:glucagon co‐infusion significantly increased heart rate as well as the rate pressure product, an indirect measure of cardiac work and myocardial oxygen consumption. Rate pressure product is an independent predictor of both in‐hospital cardiac mortality and long‐term all‐cause mortality in patients with acute coronary syndrome. The increase in the rate pressure product in our study was driven by an increase in heart rate rather than systolic BP. Increases in heart rate due to native GLP‐1 and glucagon co‐infusion^18^ (Supporting Information Table S2) and dual GLP‐1:glucagon receptor agonists are widely published but less is known about their short‐term effects on the rate pressure product. This increase in the rate pressure product seen with co‐infusion may be consistent with long‐acting GLP‐1:glucagon dual receptor agonists in the initial period after starting therapy. Reassuringly, any possible short‐term increase in the rate pressure product is shown to be ameliorated over time due to the beneficial effects on BP.^35^ Previous concerns were raised in this field over whether the increase in heart rate may negatively affect those with diastolic dysfunction, cardiomyopathy and heart failure. Clinical trial data examining the effect of GLP‐1:glucagon dual receptor agonists on heart failure related outcomes are awaited.

Haemodynamic data on GLP‐1:glucagon dual receptor agonists in clinical development are available for several compounds, including cotadutide, survodutide and pemvidutide. Cotadutide (MEDI0382), a GLP:glucagon receptor dual agonist with a ratio of approximately 5:1 GLP‐1 to glucagon activity, increases heart rate by 5 bpm (*P* < .001) in patients with type 2 diabetes and chronic kidney disease.^36^ Similar findings are reported in patients with type 2 diabetes and obesity (mean heart rate +12 bpm, systolic BP −10 mmHg).^37^ Survodutide (BI 456906) increases heart rate by 1‐6 bpm in patients with type 2 diabetes and is currently being tested in a phase 3 cardiovascular outcome trial in adults with obesity and increased cardiovascular risk.^38^, ^39^ Pemvidutide (ALT‐801) reduces systolic and diastolic BP without leading to any clinically significant increase in heart rate.^35^

From a metabolic perspective, the metabolic changes in all infusion arms align with previous published studies confirming GLP‐1 and glucagon receptor engagement. It was not possible to measure exenatide concentrations but note is made of supraphysiological glucagon levels in Part A (mean at 120 min 1945 ± 328 pg/mL) and Part B (mean at 30 min 899 ± 59 pg/mL). It is evident that glucagon offsets the glucose lowering efficacy of exenatide in this study. This highlights the importance of the respective ratios of GLP‐1:glucagon dual agonist compounds for future treatments in metabolic populations to avoid any deleterious effects.

### 4.4 Limitations

This was a small exploratory pilot study meaning it is not possible to draw firm conclusions. Only healthy adult males under 40 years old were eligible to participate. Females, older adults and those with chronic conditions, including type 2 diabetes, were excluded, meaning the current findings may not necessarily apply in other clinical settings. Stroke volume and cardiac output were measured using non‐invasive devices. Although these devices are validated, catheter‐based testing methods (ie thermodilution method, Fick's method) remain the gold standard. Adverse events, in particular nausea and vomiting, may have affected haemodynamic parameters. Observed haemodynamic effects may have also been affected by other hormones, including insulin. The infusion duration was short and therefore it is not possible to extrapolate the cardiovascular and metabolic outcomes over a longer duration, and even less so in the case of a purported future chronic oral treatment. GLP‐1 and glucagon receptor occupancy and activation differ compared to molecules in clinical development, meaning caution should be applied when making direct comparisons. Finally, although participants were blinded to the infusion, the study team was unblinded.

## 5 CONCLUSION

A short‐term co‐infusion of exenatide:glucagon increases the rate pressure product. This is driven by an increase in heart rate rather than by an increase in systolic BP. Further work is required to explore how these findings compare with dual GLP‐1:glucagon receptor agonists in clinical development and how this affects cardiovascular outcomes in those with heart failure.

### Author Contribution Statement

J.G., V.P., C.M.M., L.J., J.C., P.A. and I.B.W. conceived and designed the study. I.B.W. acted as the chief investigator for the study. J.G., G.D.S., A.H., E.V., J.H. and F.K. performed all study visits. N.J. performed all echocardiograms. P.B. analysed bloods. J.G., V.P., C.M.M., L.J., J.C., P.A. and I.B.W. analysed and interpreted the data. J.G. wrote the first draft of the manuscript. All authors critically revised the manuscript and approved the final version. The authors confirm that the Principal Investigator for this paper is I.B.W. and that he had direct clinical responsibility for patients.

### Conflict of Interest Disclosures

V.P., L.J. and P.A. are employees and shareholders of AstraZeneca.

## Supporting information


**SUPPORTING INFORMATION FIGURE S1** Stroke volume and heart rate (Part A).
**SUPPORTING INFORMATION FIGURE S2** Cardiac output and peripheral vascular resistance (Part A).
**SUPPORTING INFORMATION FIGURE S3** Brachial systolic and diastolic blood pressure (Part A).
**SUPPORTING INFORMATION FIGURE S4** Central systolic blood pressure and mean arterial pressure (Part A).
**SUPPORTING INFORMATION FIGURE S5** Augmentation index and heart rate variability (Part A).
**SUPPORTING INFORMATION FIGURE S6** Glucagon (Part A).
**SUPPORTING INFORMATION FIGURE S7** Stroke volume and heart rate (Part B).
**SUPPORTING INFORMATION FIGURE S8** Cardiac output and peripheral vascular resistance (Part B).
**SUPPORTING INFORMATION FIGURE S9** Brachial systolic and diastolic blood pressure (Part B).
**SUPPORTING INFORMATION FIGURE S10** Central systolic blood pressure and mean arterial pressure (Part B).
**SUPPORTING INFORMATION FIGURE S11** Rate pressure product (Part B).
**SUPPORTING INFORMATION FIGURE S12** Augmentation index and heart rate variability (Part B).
**SUPPORTING INFORMATION FIGURE S13** Free fatty acids and triglycerides (Part B).
**SUPPORTING INFORMATION FIGURE S14** Total GLP‐1 and total active GLP‐1 (Part B).
**SUPPORTING INFORMATION FIGURE S15** Gastric inhibitory polypeptide (glucose‐dependent insulinotropic polypeptide) and peptide Y‐Y (Part B).
**SUPPORTING INFORMATION TABLE S1** Haemodynamic effects of intravenous exenatide in humans.
**SUPPORTING INFORMATION TABLE S2** Haemodynamic and metabolic effects of native GLP‐1 and glucagon co‐infusion studies in humans

## Data Availability

The data that support the findings of this study are available from the corresponding author upon reasonable request.
