# Supplementary material for: An experimental medicine protocol for exploring the haemodynamic effects of dual agonism at the glucagon‐like peptide‐1 and glucagon receptor in healthy subjects
Source: Br J Clin Pharmacol. 2025 Sep 30;92(2):579–88. doi: 10.1002/bcp.70282 (PMC12850551; doi:10.1002/bcp.70282)
Supplement: Supplementary file 1 — SUPPORTING INFORMATION FIGURE S1 Stroke volume and heart rate (Part A). SUPPORTING INFORMATION FIGURE S2 Cardiac output and peripheral vascular resistance (Part A). SUPPORTING INFORMATION FIGURE S3 Brachial systolic and diastolic blood pressure (Part A). SUPPORTING INFORMATION FIGURE S4 Central systolic blood pressure and mean arterial pressure (Part A). SUPPORTING INFORMATION FIGURE S5 Augmentation index and heart rate variability (Part A). SUPPORTING INFORMATION FIGURE S6 Glucagon (Part A). SUPPORTING INFORMATION FIGURE S7 Stroke volume and heart rate (Part B). SUPPORTING INFORMATION FIGURE S8 Cardiac output and peripheral vascular resistance (Part B). SUPPORTING INFORMATION FIGURE S9 Brachial systolic and diastolic blood pressure (Part B). SUPPORTING INFORMATION FIGURE S10 Central systolic blood pressure and mean arterial pressure (Part B). SUPPORTING INFORMATION FIGURE S11 Rate pressure product (Part B). SUPPORTING INFORMATION FIGURE S12 Augmentation index and heart rate variability (Part B). SUPPORTING INFORMATION FIGURE S13 Free fatty acids and triglycerides (Part B). SUPPORTING INFORMATION FIGURE S14 Total GLP‐1 and total active GLP‐1 (Part B). SUPPORTING INFORMATION FIGURE S15 Gastric inhibitory polypeptide (glucose‐dependent insulinotropic polypeptide) and peptide Y‐Y (Part B). SUPPORTING INFORMATION TABLE S1 Haemodynamic effects of intravenous exenatide in humans. SUPPORTING INFORMATION TABLE S2 Haemodynamic and metabolic effects of native GLP‐1 and glucagon co‐infusion studies in humans [file BCP-92-579-s001.pdf]

Supplementary appendix

Contents

Supplementary Figure 1: Heart rate and stroke volume (Part A)....3

Supplementary Figure 2: Cardiac output and peripheral vascular resistance (Part A) 4

Supplementary Figure 3: Brachial systolic and diastolic blood pressure (Part A)....5

Supplementary Figure 4: Central systolic blood pressure and mean arterial pressure (Part A)....6

Supplementary Figure 5: Augmentation index and heart rate variability (Part A) 7

Supplementary Figure 6: Glucagon (Part A) 8

Supplementary Figure 7: Stroke volume and heart rate (Part B) 9

Supplementary Figure 8: Cardiac output and peripheral vascular resistance (Part B)....10

Supplementary Figure 9: Brachial systolic and diastolic blood pressure (Part B)....11

Supplementary Figure 10: Central systolic blood pressure and mean arterial pressure (Part B)....12

Supplementary Figure 11: Rate pressure product (Part B) 13

Supplementary Figure 12: Augmentation index and heart rate variability (Part B) 14

Supplementary Figure 13: Free fatty acids and triglycerides (Part B)....15

Supplementary Figure 14: Total GLP-1 and total active GLP-1 (Part B)....16

Supplementary Figure 15: Gastric inhibitory polypeptide (glucose-dependent insulinotropic polypeptide) and Peptide Y-Y (Part B)....17

Supplementary Table 1: Haemodynamic effects of intravenous exenatide in humans ……………..18

Supplementary Table 2: Haemodynamic and metabolic effects of native GLP-1 and glucagon co-infusion studies in humans……20

Supplementary Figure 1: Stroke volume and heart rate (Part A)

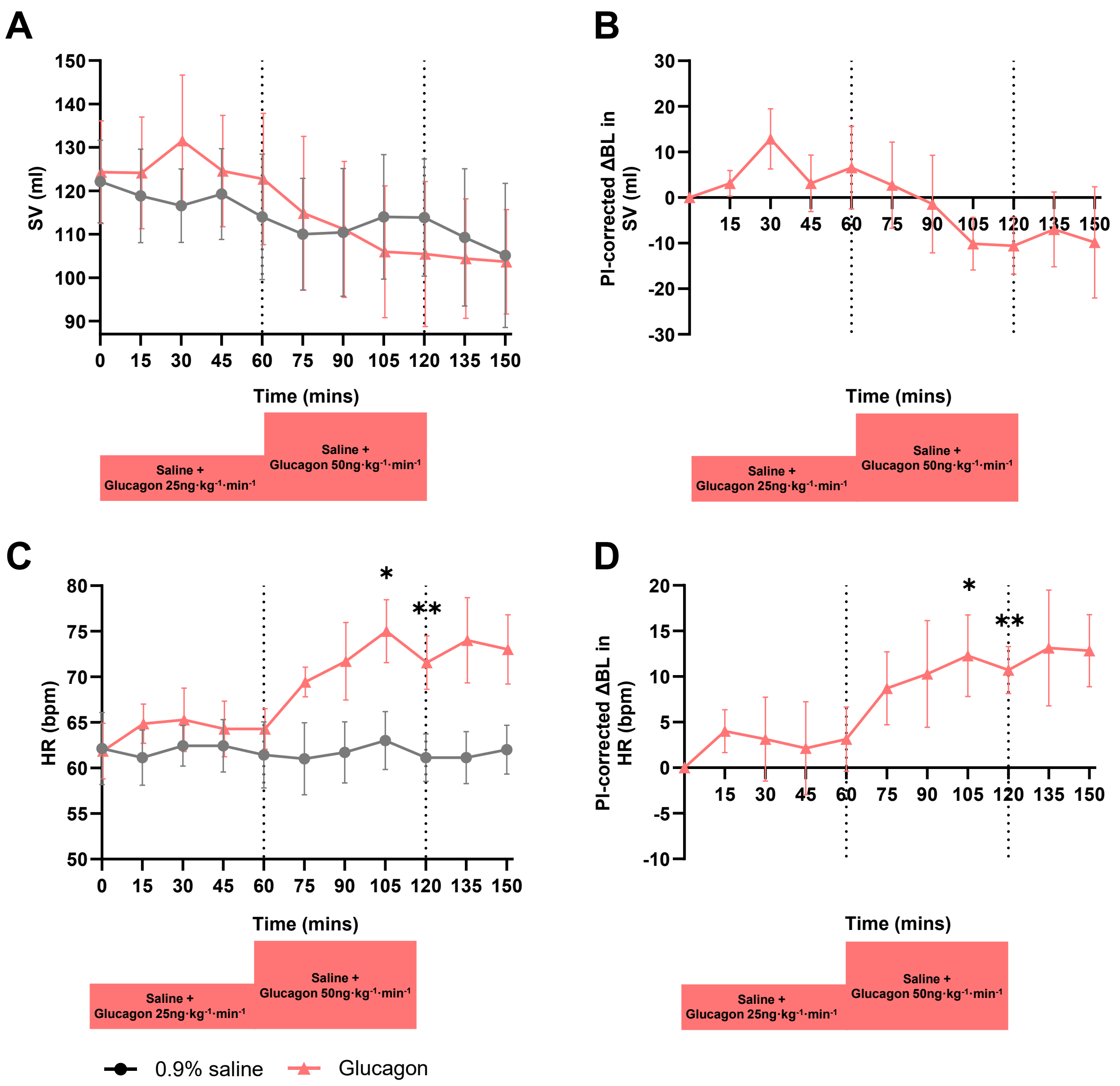

A – SV (stroke volume). B – stroke volume, placebo corrected change from baseline. C – HR (heart rate). D – heart rate, placebo corrected change from baseline.
Data are unadjusted means ± SEM. *p<0.05, **p<0.01, ***p<0.001

Supplementary Figure 2: Cardiac output and peripheral vascular resistance (Part A)

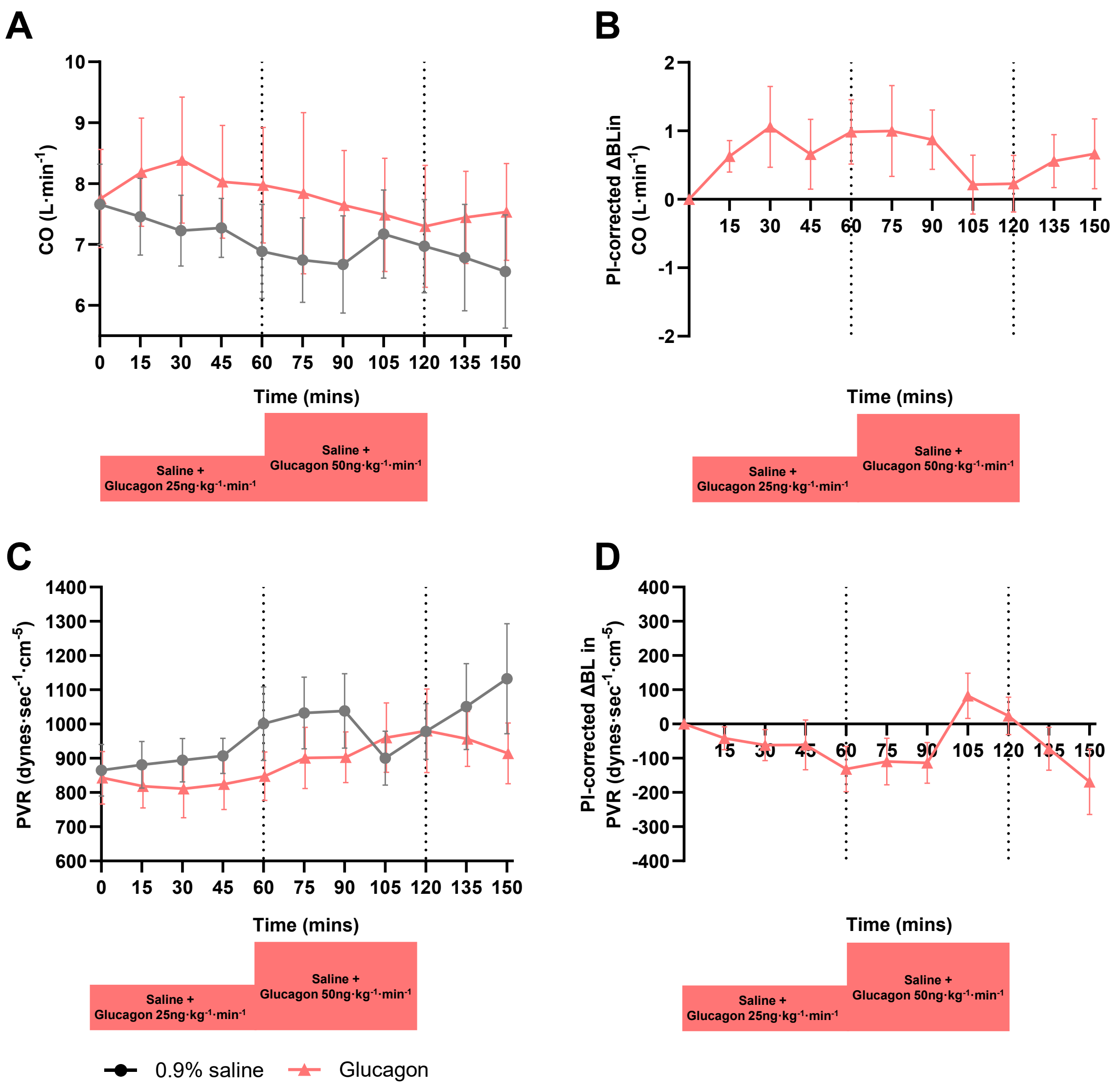

A – CO (cardiac output). B – cardiac output, placebo corrected change from baseline. C – PVR (peripheral vascular resistance). D – PVR, placebo corrected change from baseline.
Data are unadjusted means ± SEM.

Supplementary Figure 3: Brachial systolic and diastolic blood pressure (Part A)

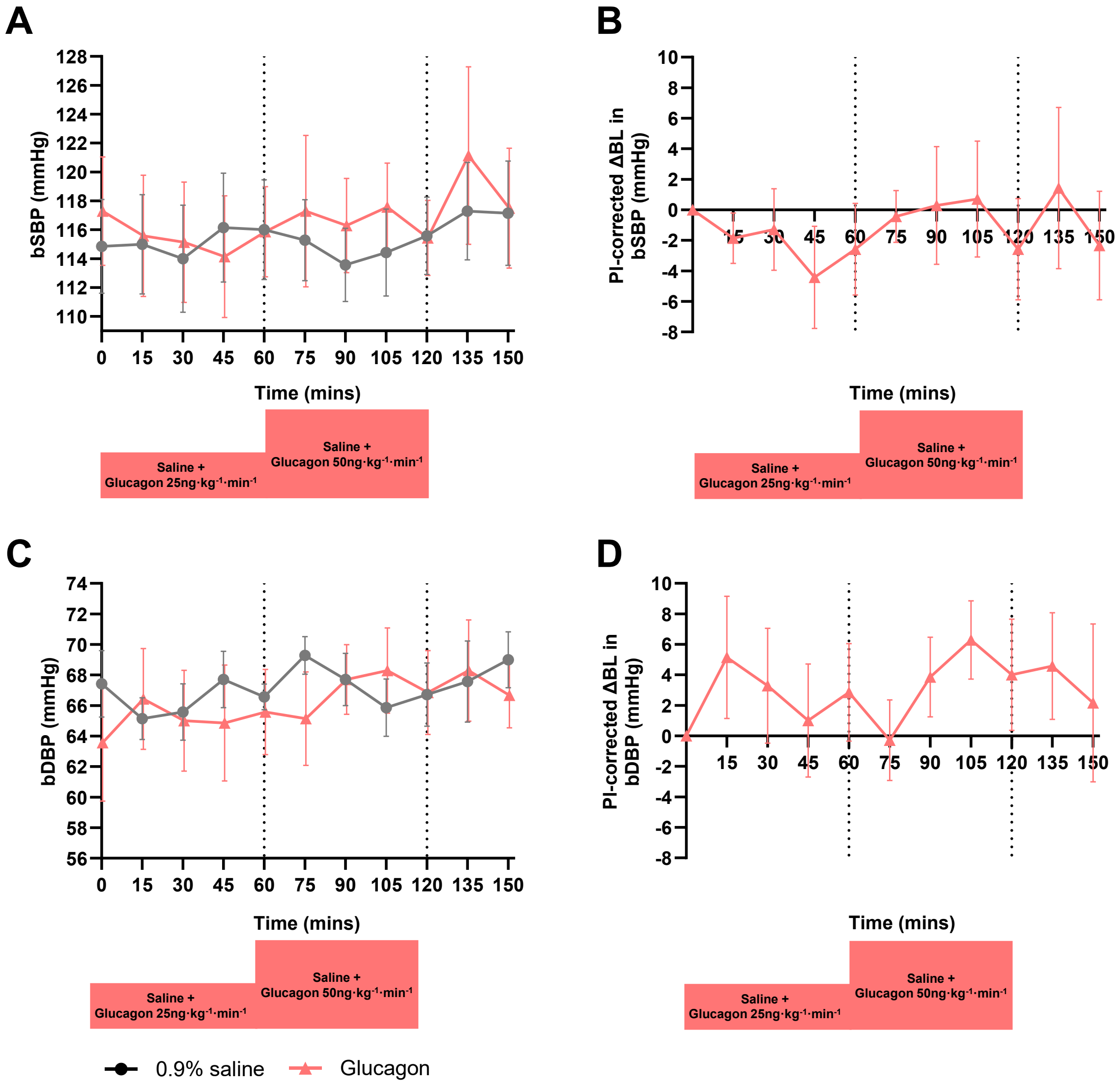

A – bSBP (brachial systolic blood pressure). B – bSBP, placebo corrected change from baseline. C – bDBP (brachial diastolic blood pressure). D – bDBP, placebo corrected change from baseline.

Data are unadjusted means ± SEM.

Supplementary Figure 4: Central systolic blood pressure and mean arterial pressure (Part A)

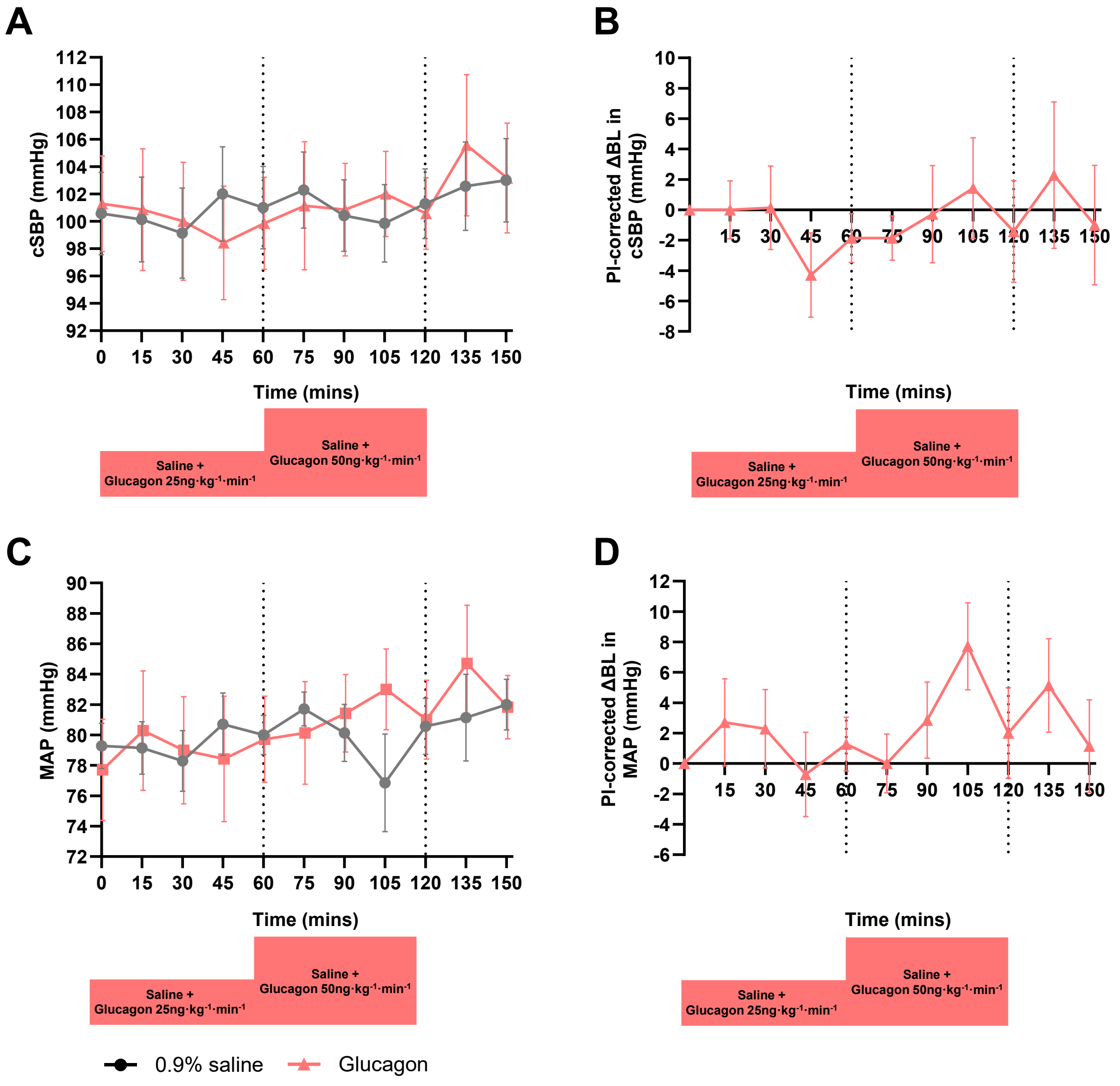

A – cSBP (central systolic blood pressure). B – cSBP, placebo corrected change from baseline. C – MAP (mean arterial pressure). D – MAP, placebo corrected change from baseline. Data are unadjusted means ± SEM.

Supplementary Figure 5: Augmentation index and heart rate variability (Part A)

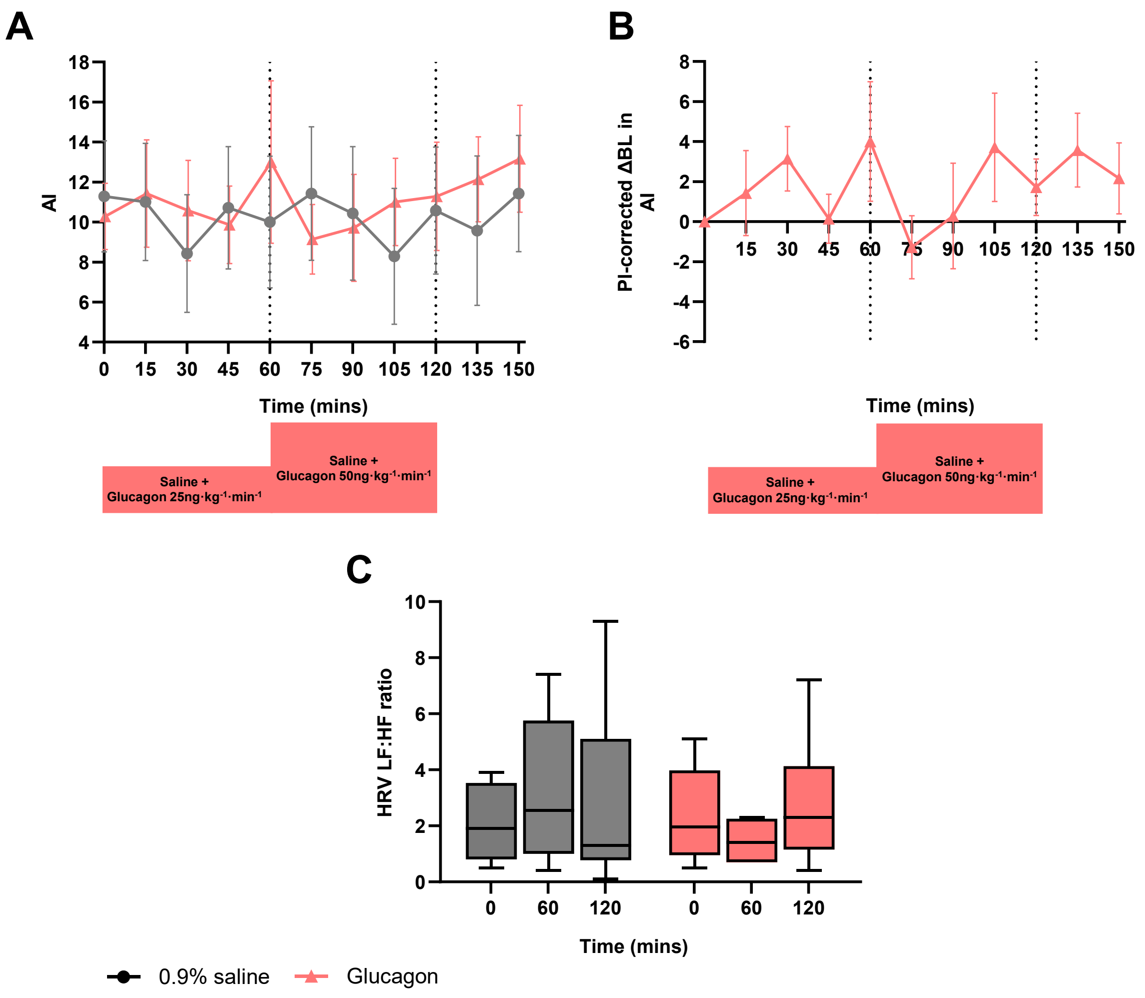

A – AI (augmentation index). B – AI, placebo corrected change from baseline. C – HRV LF:HF (heart rate variability low frequency:high frequency).
Data are unadjusted means ± SEM.

Supplementary Figure 6: Glucagon (Part A)

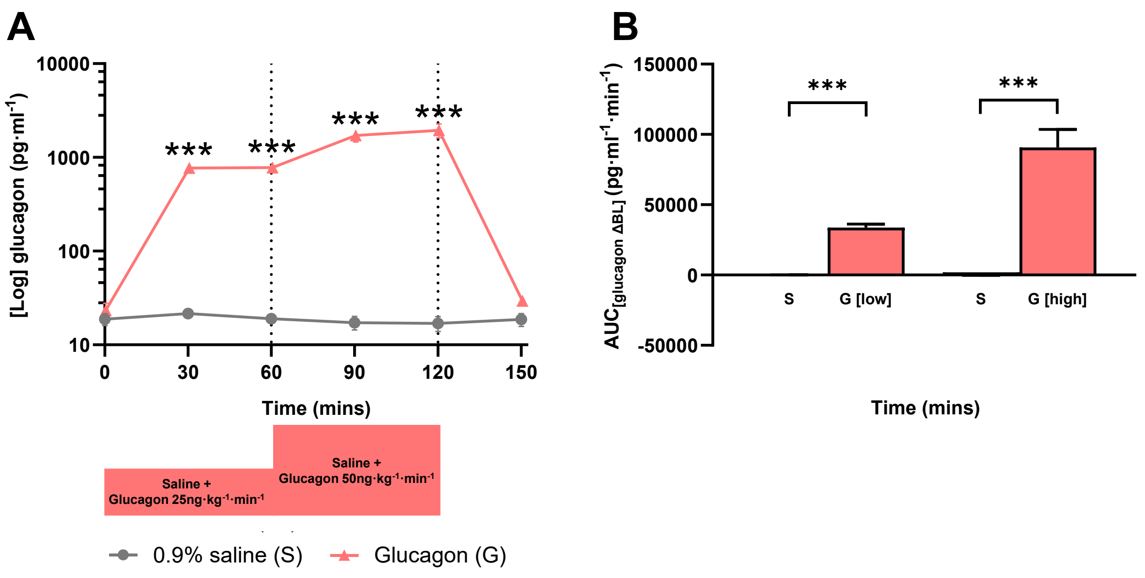

A – glucagon. B – glucagon, change from baseline, presented as area under the concentration-time curve (0-120 min).
Glucagon = infused (synthetic) glucagon + native glucagon
Data are unadjusted means ± SEM. *p<0.05, **p<0.01, ***p<0.001

Supplementary Figure 7: Stroke volume and heart rate (Part B)

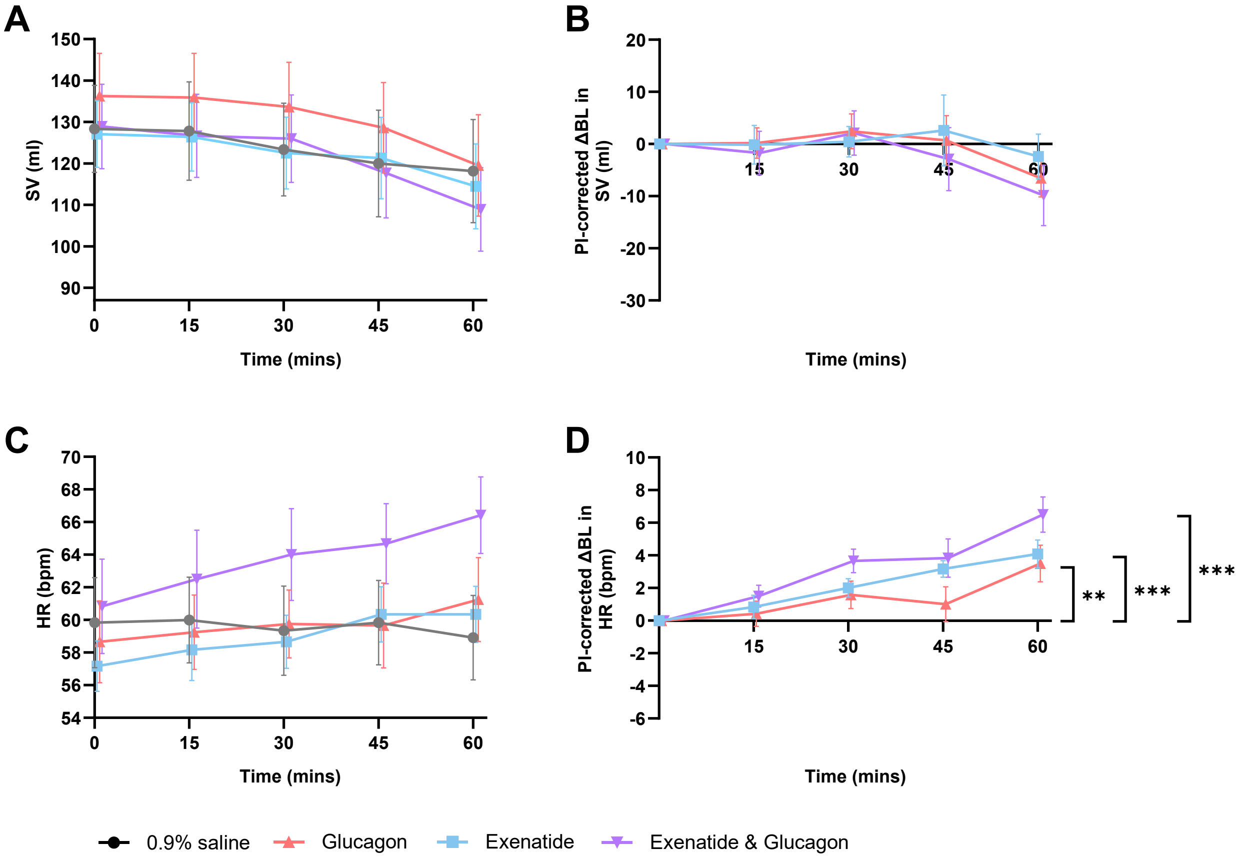

A – SV (stroke volume). B – stroke volume, placebo corrected change from baseline. C – HR (heart rate). D – heart rate, placebo corrected change from baseline.
Data are unadjusted means ± SEM. *$p<0.05$, **$p<0.01$, ***$p<0.001$

Supplementary Figure 8: Cardiac output and peripheral vascular resistance (Part B)

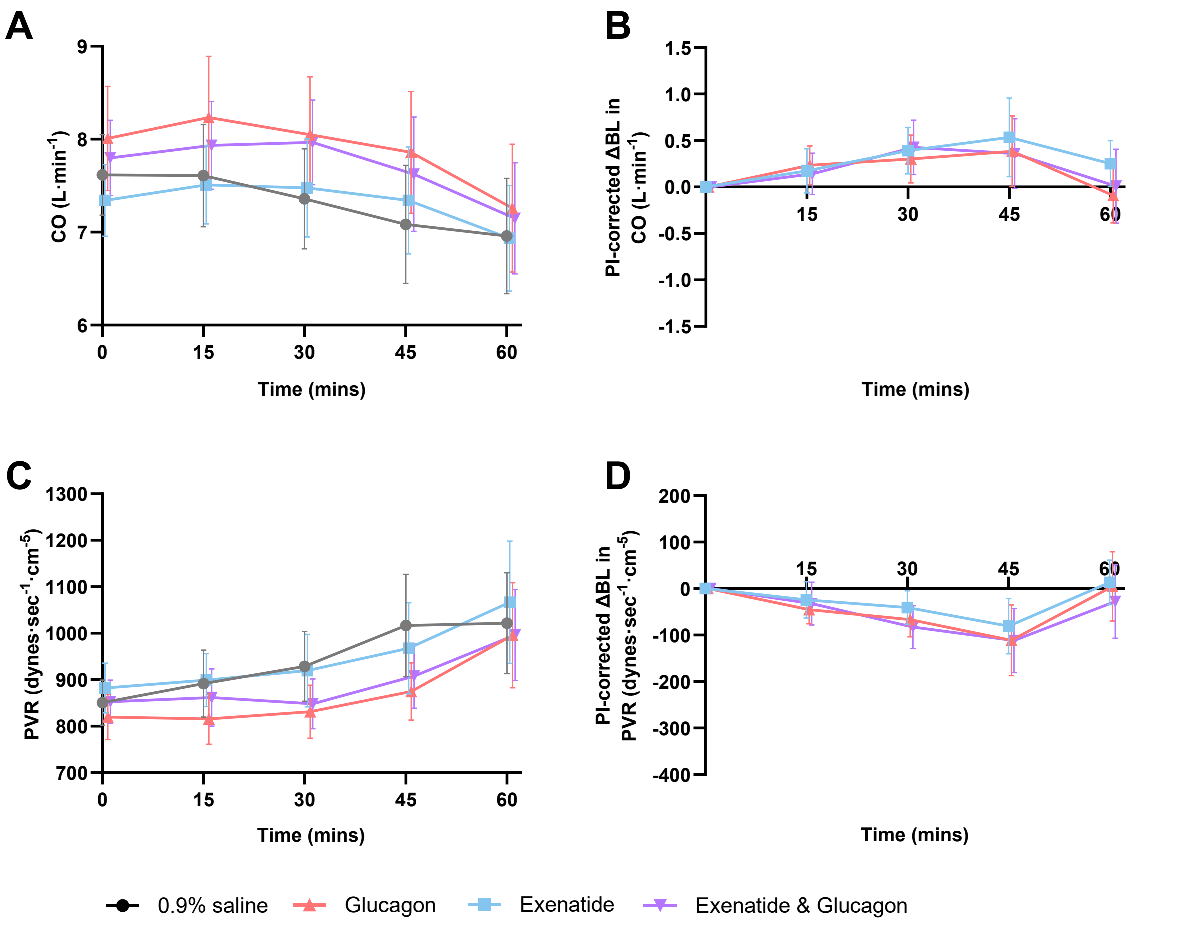

A – CO (cardiac output). B – cardiac output, placebo corrected change from baseline. C – PVR (peripheral vascular resistance). D – PVR, change from baseline.
Data are unadjusted means ± SEM.

Supplementary Figure 9: Brachial systolic and diastolic blood pressure (Part B)

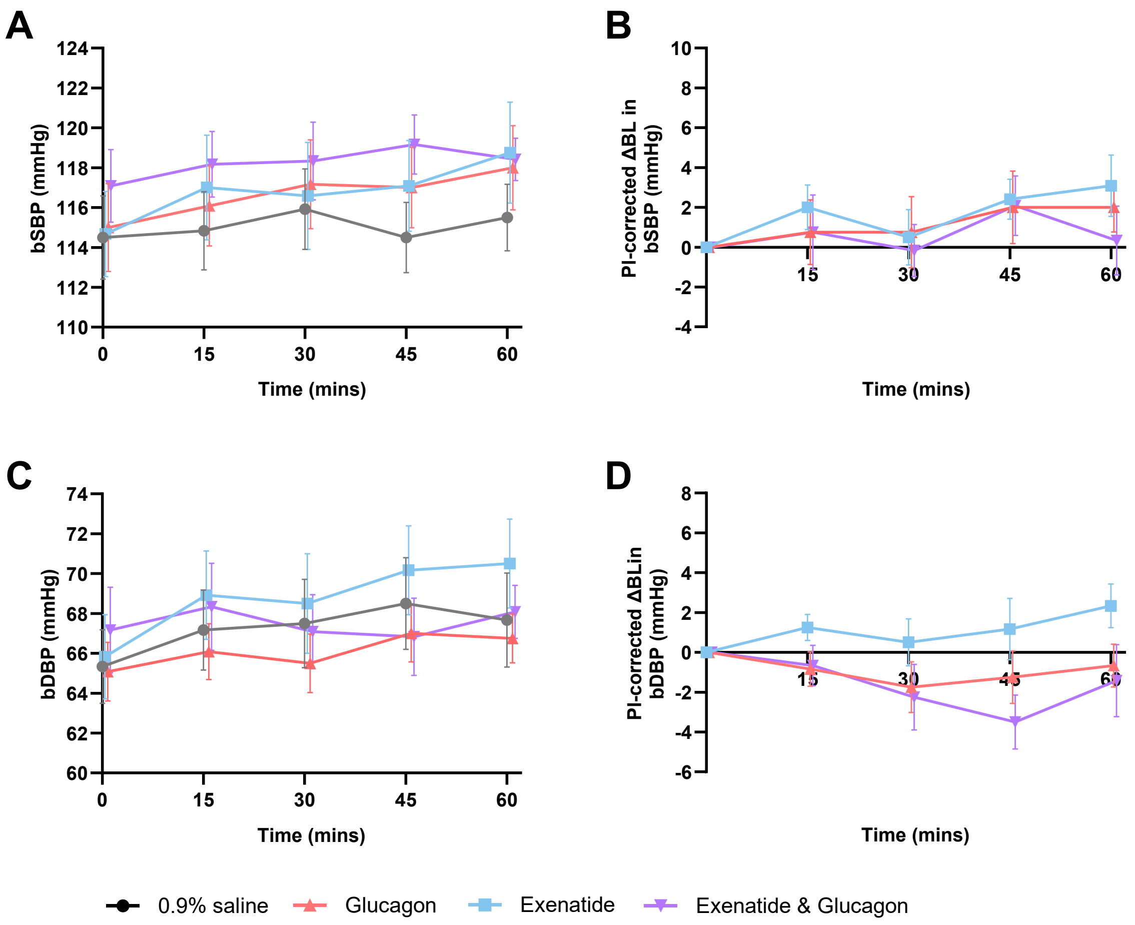

A – bSBP (brachial systolic blood pressure). B – bSBP, placebo corrected change from baseline. C – bDBP (brachial diastolic blood pressure). D – bDBP, placebo corrected change from baseline.

Data are unadjusted means ± SEM.

Supplementary Figure 10: Central systolic blood pressure and mean arterial pressure (Part B)

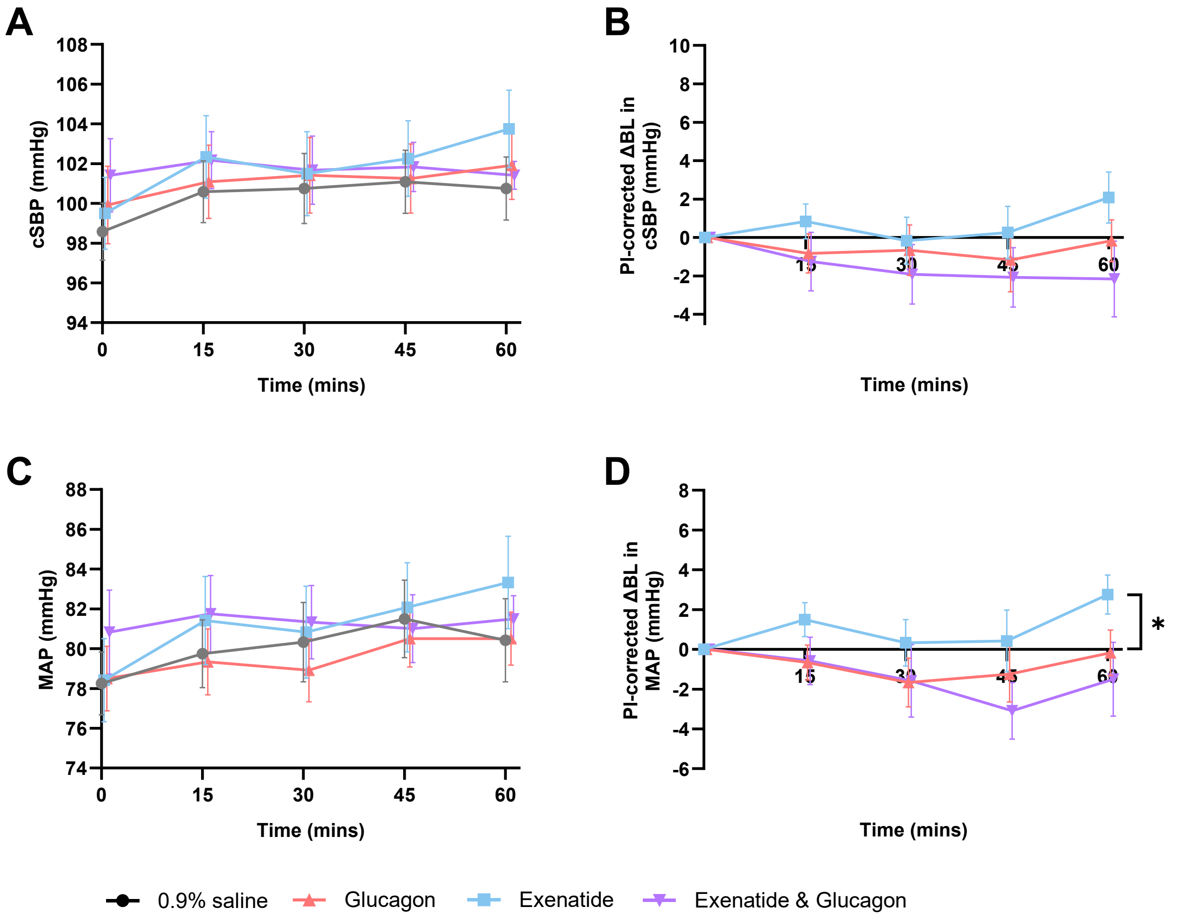

A – cSBP (central systolic blood pressure). B – cSBP, placebo corrected change from baseline.
C – MAP (mean arterial pressure). D – MAP, placebo corrected change from baseline.
Data are unadjusted means ± SEM. *p<0.05

Supplementary Figure 11: Rate pressure product (Part B)

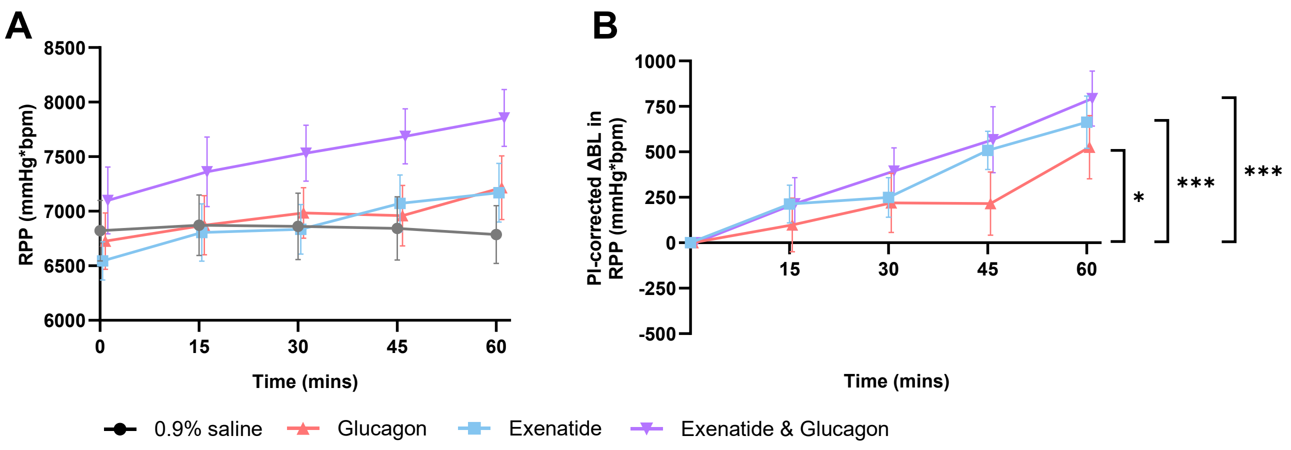

A – RPP (rate pressure product). B – RPP, change from baseline.
Data are unadjusted means ± SEM. *$p<0.05$, **$p<0.01$, ***$p<0.001$

Supplementary Figure 12: Augmentation index and heart rate variability (Part B)

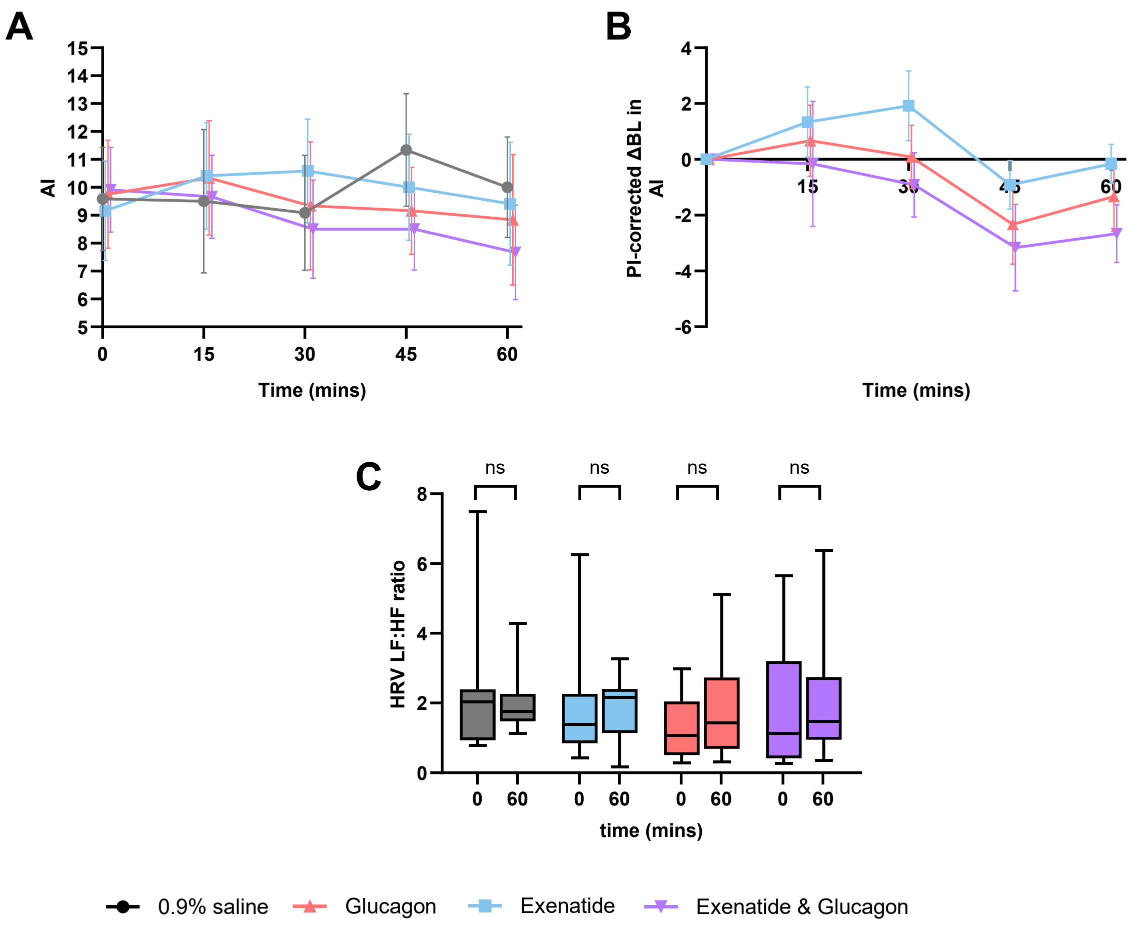

A – AI (augmentation index). B – AI, change from baseline. C – HRV LF:HF (heart rate variability low frequency:high frequency).
Data are unadjusted means ± SEM

Supplementary Figure 13: Free fatty acids and triglycerides (Part B)

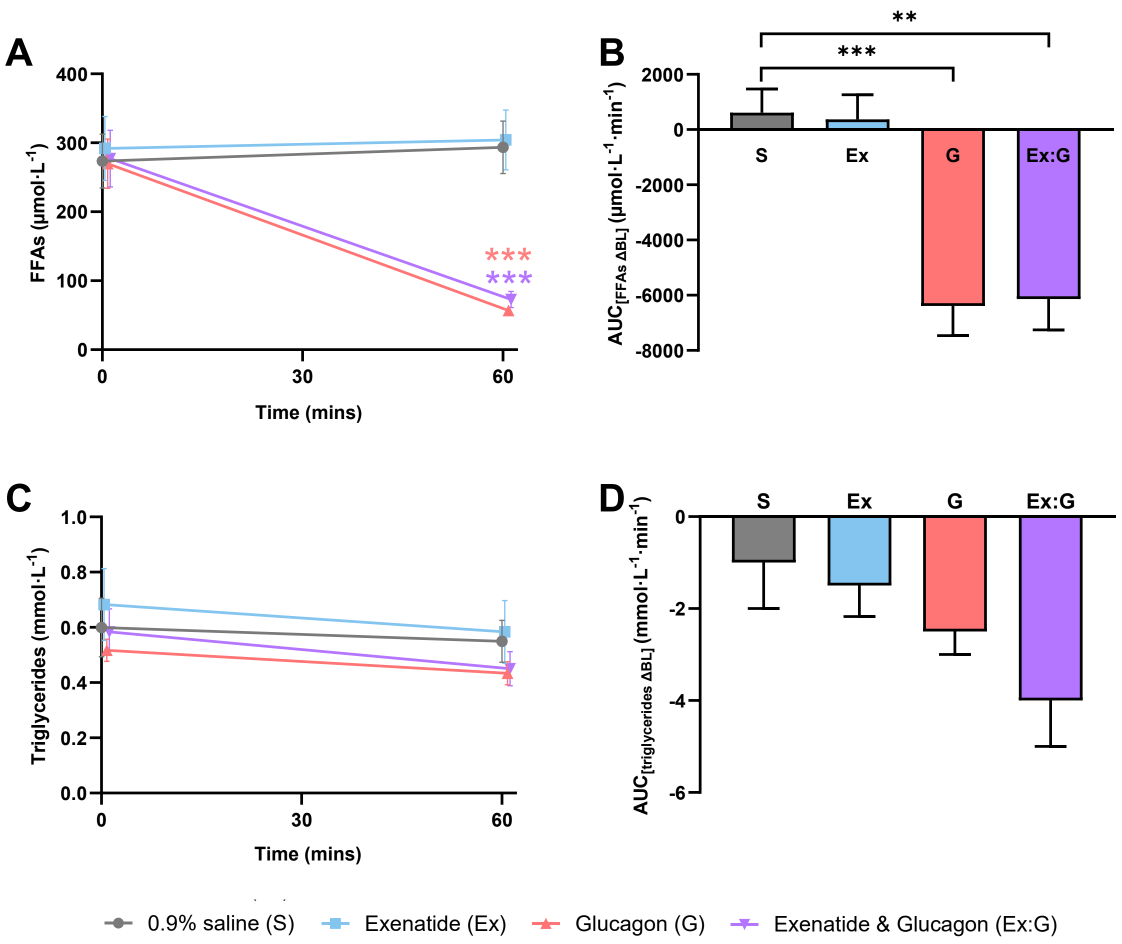

A – FFAs (free fatty acids). B – FFAs, change from baseline presented as area under the curve (0-60 min). C – triglycerides. D – C-triglycerides, change from baseline presented as area under the curve (0-60 min).

Data are unadjusted means ± SEM. *$p<0.05$, **$p<0.01$, ***$p<0.001$

Supplementary Figure 14: Total GLP-1 and total active GLP-1 (Part B)

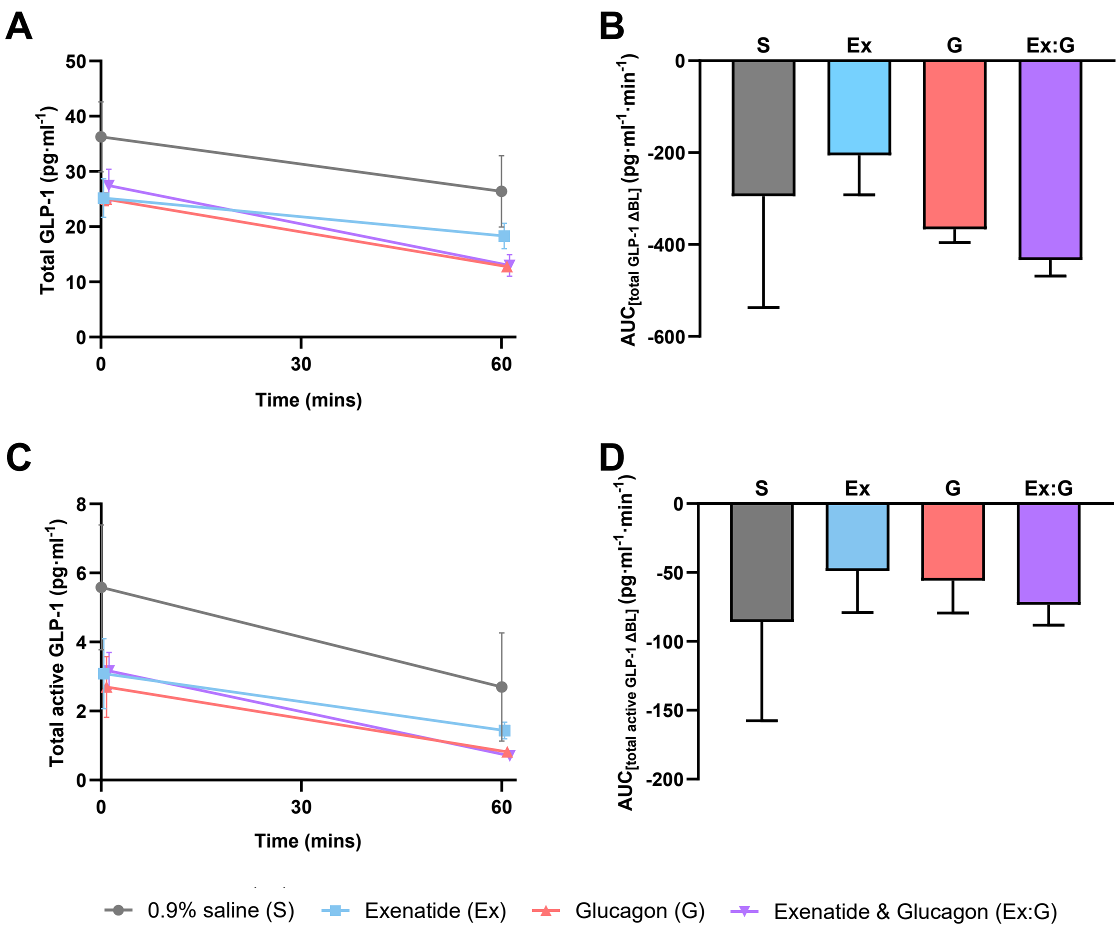

A – Total GLP-1. B – Total GLP-1, change from baseline presented as area under the curve (0-60 min). C – total active GLP-1. D – Total active GLP-1, change from baseline presented as area under the curve (0-60 min).
Data are unadjusted means ± SEM.

Supplementary Figure 15: Gastric inhibitory polypeptide (glucose-dependent insulinotropic polypeptide) and Peptide Y-Y (Part B)

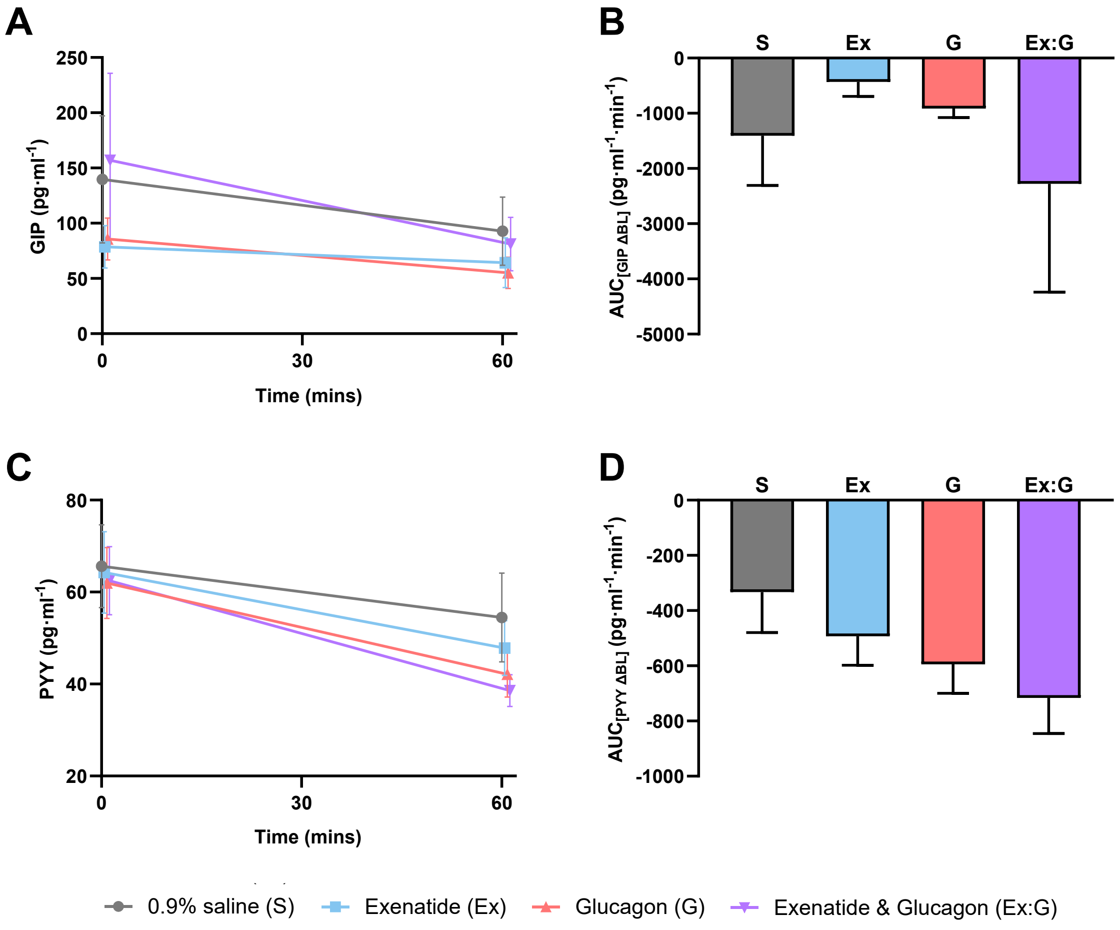

A – GIP (Gastric inhibitory polypeptide / glucose-dependent insulinotropic polypeptide). B – GIP, change from baseline presented as area under the curve (0-60 min). C – PYY (peptide Y-Y). D – PYY, change from baseline presented as area under the curve (0-60 min).
Data are unadjusted means ± SEM.

Supplementary Table 1: Haemodynamic effects of intravenous exenatide in humans

**Study**	**Population**	**Study drugs**	**Exenatide dose** ng/kg/min (pmol/kg/min)	**Duration** Minutes (m) Hours (h)	**Cardiovascular findings**
Nathanson et al[1,2], 2012	20 males<br>T2DM and heart failure	exenatide<br>placebo	0.50 ng/kg/min (0.12 pmol/kg/min)	6 h	Exenatide increased CI (0.3 L/min/m^2, 17% at 6h), CO (0.7 L/min, 18%) and HR (21 bpm, 9% at 6h). Exenatide decreased RAP, PCWP and ANP. No effect on SV, SBP, DBP, MAP, PVR, NT-pro BNP.
Darpo et al[3] 2013	11 healthy participants	exenatide<br>saline<br>moxifloxacin	Target exenatide concentrations of 200 pg ml^{-1} on D1, 300 pg ml^{-1} on D2, 500 pg ml^{-1} on D3	67 h	Exenatide increased HR (16.8 bpm). No prolongation of the QTcP >450ms or change from baseline >30ms. No effects on PR or QRS interval.
Smits et al[4] 2015	10 males<br>elevated BMI	exenatide<br>saline<br>L-MNNA<br>L-NMMA:exenatide	[a]50 ng/min for 30m then 25 ng/min	60 m	Exenatide increased HR by 5 bpm and microvascular perfusion (capillary density measured using capillary videomicroscopy). No change in SBP or DBP.
Muskiet et al[5] 2016	10 males<br>elevated BMI	Exenatide<br>saline<br>L-NMMA<br>L-NMMA:exenatide	[a]50 ng/min for 30m then 25 ng/min	120 m	Exenatide increased GFR, ERPF, glomerular pressure and HR. No change in SBP, DBP or MAP.
Smits et al[6] 2016	10 males<br>elevated BMI	exenatide<br>saline<br>L-NMMA<br>L-NMMA:exenatide	[a]50 ng/min for 30m then 25 ng/min	90 m	Exenatide increased HR (7 bpm), SBP (10 mmHg), CO (1.3 L/min) and markers of SNS activity (median LH:HF ratio and mean RRP). Co-infusion with L-NMMA (to ameliorate NO-induce vasodilation) failed to reduce HR, SBP or LF:HR ratio. No effect on DBP, MAP, PVR or SV compared with saline.
Smits et al[7] 2016	57 participants<br>T2DM	exenatide<br>saline	[a]50 ng/min for 30m then 25 ng/min	300 m	Exenatide increased SBP (7.3 mm Hg), DBP (2.9 mm Hg) and HR (4.8 bpm) compared with saline. Exenatide increased fasting skin SNS activity and postprandial

					endothelial function. No effect on capillary perfusion in the fasting or post-prandial state.
Cirincione et al[8] 2017	21 healthy participants	exenatide	1.0 to 6.3 μg/h, in order to achieve average target steady-state concentrations of 200, 300, 500, and 700 pg/mL on days 1, 2, 3, and 4, respectively.	4 days	HR increased from 65 bpm (baseline) to 74 bpm, 77 bpm, and 80 bpm with increasing exenatide concentrations on days 2, 3, and 4, respectively.
Smits et al[9] 2017	57 participants T2DM	exenatide<br>saline<br>liraglutide	[a]50 ng/min for 30m then 25 ng/min	150 m	Exenatide increased HR, SBP, DBP, PVR, SDNN, and RMSSD. Stroke volume and AI decreased. No effect on LF:HF ratio or CO.<br>Liraglutide increased HR and decreased SBP and SVI. No effect on SNS activity. Diastolic BP, MAP, CI, PVR and AI unchanged.

AI; augmentation index; ANP, atrial natriuretic peptide; CI, cardiac index; CO, cardiac output; DBP; diastolic blood pressure; ERPF, effective *renal* plasma flow; GFR, glomerular filtration rate; HF, high-frequency; HR, heart rate; LF, low-frequency; MAP, mean arterial pressure; NEFA, non-esterified fatty acids; NO, nitric oxide; NT-pro BNP, N-terminal pro b-type natriuretic peptide; PCWP, pulmonary capillary web pressure; PVR, peripheral vascular resistance; RAP, right atrial pressure; RMSSD, root mean square of successive difference between normal heartbeats; SBP, systolic blood pressure; SDNN, standard deviation of normal-normal R-R intervals; SNS, sympathetic nervous system; SV, stroke volume; SVI, stroke volume index; T2DM, type 2 diabetes mellitus

Supplementary Table 2: Haemodynamic and metabolic effects of native GLP-1 and glucagon co-infusion studies in humans

Study	Population	Study drugs	Duration min	Glucagon ng/kg/min (pmol/kg/min)	GLP-1 pmol/kg/min	Metabolic findings	Cardiovascular findings[b]
Tan et al[10] 2013	10 Elevated BMI	glucagon<br>GLP-1<br>GLP-1:glucagon<br>placebo (gelufusine)	45	50 (14.4)	0.8	Dual agonism increases energy expenditure, blunts hyperglycaemic effect of glucagon, reduces NEFA, suppresses ghrelin	No change in brachial SBP or brachial DBP. Trend towards an increase in HR with glucagon and co-infusion
Cegla et al[11] 2014	10 Elevated BMI	glucagon<br>GLP-1<br>GLP-1:glucagon<br>placebo (gelofusine)	120[a]	9.8 (2.8)	0.4	Dual agonism Increases energy expenditure, blunts hyperglycaemic effect of glucagon, reduces food intake	No changes in SBP, DBP, HR with any of the treatment groups
Blagger et al[12] 2015	15 males	glucagon<br>GLP-1<br>GLP-1:glucagon<br>oxyntomodulin<br>placebo (0.9% saline)	240	3.0 (0.9)	1.0		No cardiovascular measurements

BMI indicates body mass index, DBP; diastolic blood pressure; HR, heart rate; MAP, mean arterial pressure; NEFA, non-esterified fatty acids; SBP, systolic blood pressure;

[a]5 min ramping at four times rate, then further 5 min ramping at two times rate.

[b]No adverse events were reported for any of the studies. CV data were not collected in the study by Blagger et al.

References

1. Nathanson, D., et al., *Effects of intravenous exenatide in type 2 diabetic patients with congestive heart failure: a double-blind, randomised controlled clinical trial of efficacy and safety.* Diabetologia, 2012. **55**(4): p. 926-35.
2. Nathanson, D., et al., *Exenatide infusion decreases atrial natriuretic peptide levels by reducing cardiac filling pressures in type 2 diabetes patients with decompensated congestive heart failure.* Diabetology & Metabolic Syndrome, 2016. **8**(1): p. 5.
3. Darpo, B., et al., *Exenatide at therapeutic and supratherapeutic concentrations does not prolong the QTc interval in healthy subjects.* Br J Clin Pharmacol, 2013. **75**(4): p. 979-89.
4. Smits, M.M., et al., *GLP-1 Receptor Agonist Exenatide Increases Capillary Perfusion Independent of Nitric Oxide in Healthy Overweight Men.* Arterioscler Thromb Vasc Biol, 2015. **35**(6): p. 1538-43.
5. Muskiet, M.H., et al., *Acute renal haemodynamic effects of glucagon-like peptide-1 receptor agonist exenatide in healthy overweight men.* Diabetes Obes Metab, 2016. **18**(2): p. 178-85.
6. Smits, M.M., et al., *Exenatide acutely increases heart rate in parallel with augmented sympathetic nervous system activation in healthy overweight males.* Br J Clin Pharmacol, 2016. **81**(4): p. 613-20.
7. Smits, M.M., et al., *GLP-1-Based Therapies Have No Microvascular Effects in Type 2 Diabetes Mellitus: An Acute and 12-Week Randomized, Double-Blind, Placebo-Controlled Trial.* Arterioscler Thromb Vasc Biol, 2016. **36**(10): p. 2125-32.
8. Cirincione, B., et al., *Model-Based Evaluation of Exenatide Effects on the QT Interval in Healthy Subjects Following Continuous IV Infusion.* The Journal of Clinical Pharmacology, 2017. **57**(8): p. 956-965.
9. Smits, M.M., et al., *Heart rate acceleration with GLP-1 receptor agonists in type 2 diabetes patients: an acute and 12-week randomised, double-blind, placebo-controlled trial.* Eur J Endocrinol, 2017. **176**(1): p. 77-86.
10. Tan, T.M., et al., *Coadministration of glucagon-like peptide-1 during glucagon infusion in humans results in increased energy expenditure and amelioration of hyperglycemia.* Diabetes, 2013. **62**(4): p. 1131-8.
11. Cegla, J., et al., *Coinfusion of low-dose GLP-1 and glucagon in man results in a reduction in food intake.* Diabetes, 2014. **63**(11): p. 3711-20.
12. Bagger, J.I., et al., *Effect of Oxyntomodulin, Glucagon, GLP-1, and Combined Glucagon +GLP-1 Infusion on Food Intake, Appetite, and Resting Energy Expenditure.* J Clin Endocrinol Metab, 2015. **100**(12): p. 4541-52.
